# Computational discovery of natural inhibitors targeting enterovirus D68 3C protease using molecular docking pharmacokinetics and dynamics simulations

**DOI:** 10.1038/s41598-025-95163-y

**Published:** 2025-03-31

**Authors:** Mansoor Alsahag

**Affiliations:** 1 Faculty of Applied Medical Sciences, Al-Baha University, Al-Baha, Kingdom of Saudi Arabia; 2 Department of Clinical Infection, Microbiology and Immunology, University of Liverpool, Liverpool, United Kingdom

**Keywords:** Enterovirus D68, EV-D68, 3C protease, Antiviral therapy, Natural inhibitors, Withaferin-A, Baicalin, Molecular modelling, Dynamics simulations, Pharmacokinetics, Chemical biology, Computational biology and bioinformatics, Developmental biology, Drug discovery, Immunology, Molecular biology

## Abstract

Enterovirus D68 (EV-D68) is a significant global health threat, responsible for severe respiratory and neurological complications, with no FDA-approved antiviral treatments currently available. The 3C protease of EV-D68, an essential enzyme involved in viral replication, represents a key target for therapeutic development. In this study, we employed a comprehensive computational approach, including molecular docking, pharmacokinetic predictions, and molecular dynamics simulations, to identify potential natural inhibitors of the EV-D68 3C protease. Screening a library of natural compounds, Withaferin-A (CID: 265,237) and Baicalin (CID: 64,982) emerged as top candidates due to their favorable pharmacokinetic profiles, high binding affinities (-10.7 kcal/mol for Withaferin-A and -9.5 kcal/mol for Baicalin), and interactions with key residues in the protease’s active site. The molecular dynamics simulations demonstrated the stability of the protein–ligand complexes, with low root mean square deviation (RMSD) and fluctuation (RMSF) values over a 100-ns trajectory. Free energy calculations further supported the superior binding efficiency of Withaferin-A. These findings suggest that Withaferin-A and Baicalin have significant potential as natural inhibitors of EV-D68 3C protease, offering a promising foundation for future experimental validation and the development of targeted antiviral therapies against EV-D68.

## Introduction

Enterovirus D68 or EC-D68 belongs to family Picornaviridae, genus Enterovirus spread through direct contact or respiratory droplets. Leads to respiratory problems and other neurological problems like acute flaccid myelitis (affects the spinal cord). It consists of small icosahedral capsid which is not enveloped and composed of single stranded positive sense RNA genome^1^. Capsid is made up of 4 different proteins (VP1, VP2, VP3 and VP4) arranged in symmetrical manner with icosahedral shape, form a protective shell. RNA genome is approximately 7400 nucleotides in length that encode for polyprotein. This genome has 5’ UTR, ORF and 3’ UTR. EV-D68 first identified in 1962 after that it gain attention globally by several outbreaks in recent years specially in United States^2,3^. This virus primarily effect children and causes symptoms range from mild to severe respiratory and neurological infections. In 2014 large scale outbreak over 1100 cases across 49 different states and 120 confirmed AFM cases. Between 2016 to 2018, 186 AFM cases were reported. These massive outbreaks generally occur in different countries like Netherland, France, Italy, China, North America. Symptoms of Entrovirus-D68 can affect both respiratory and nervous systems vary from mild to severe^4^. Mild respiratory symptoms include cough, sore throat, sneezing, and runny nose. Severe symptoms include difficulty in breathing, respiratory distress, pneumonia, also require ventilation support^1^. In some cases, it shows fever, headache, fatigue. AFM or neurological systems include loss of muscle reflexes, difficulty in speaking and swallowing, sometime paralysis may occur^5^.

The structural protein includes viral protein 1 or VP1 which is the surface protein and involved in binding receptors, which are important for viruses to infect host cells. It’s a primary protein interacting with receptors, especially with CD155 it is a membrane protein act as viral receptor in some cell types^6^. Viral protein 2 and 3 are parts of viral outer envelope and stabilize the virus structure. It is not directly involved in binding the receptor. These viral proteins are important for protecting the genome of viral shells. Viral particle 4 is more involved in virus internal process and less exposed to surface like as VP1 and VP2^7,8^. VP4 play role in release of viral genome when virus enter the host cell, it released and involved in up coating process which is important for releasing of genome into the cytoplasm^7^. Non-structural proteins of Entrovirus-D68 include 2A protease, 2B and 2C proteins, 3C, 3D RdRp (RNA dependent RNA polymerase), 3’ UTR and IRES (Internal Ribosome Entry Site) these are involved in replication of viral RNA and manipulation of host machinery for replication. 2A protease cleaves the polyprotein into single functional proteins. It is the first step in the development of viruses. 2A protease inhibits the synthesis by cleaving the host protein that is involved in translation initiation. It has a catalytic site that is important for activity, disrupts the host cell machinery and gave a favor for viral replication. 2B and 2C proteins also play a role in RNA replication process, interact with host cell membrane and hijack the host cell machinery to form special compartment where replication takes place. 3C protease cleaves viral polyprotein at different sites to make a mature viral particle. It also plays a role in suppressing antiviral responses of host cells. 3D RdRp is responsible for synthesizing new RNA genome^9^. 3D polymerase has standard RNA-dependent RNA polymerase folds, which allows nucleotides to add in RNA chain^10,11^. It is a beneficial enzyme for viral replication. 3’UTR and ribosome entry sites are not proteins but play a vital role in translation and replication of viral RNA. IRES allows the virus to stop or catch the ribosome and start translation of host normal cap dependent translation mechanism^12,13^.

Entrovirus-D68 infects the respiratory tract, main function is to bind with the host cell, entry and hijack the host machinery to assemble and replicate the new virus particle. Entry with the help of receptor known as CD155 (poliovirus receptor)^14^. After that it enters the cell by receptor mediated endocytosis method, in which virus particles engulf within the vesicles, then viral RNA genome releases the virus particles into the cytoplasm and then translation process starts^13^. During replication and assembly process the RNA genome translated into large polyprotein and split into different proteins by viral protease including both structural and non-structural proteins. Non-structural proteins play a role in replication, assembly and transcription of viruses^15^. Among the non-structural proteins of EV-D68, the 3C protease is a particularly attractive drug target due to its crucial role in viral replication and immune evasion. Unlike 2A protease, which primarily cleaves host proteins to disrupt host translation, 3C protease is responsible for processing the viral polyprotein into functional units, making it indispensable for viral maturation^16^. Additionally, compared to 3D RNA-dependent RNA polymerase (3D RdRp), which is a conventional target for nucleoside analogs, 3C protease presents a more structurally defined and druggable binding pocket, as demonstrated in previous studies on EV-A71 and CVB3 protease inhibitors. Given its sequence conservation across enteroviruses and lack of FDA-approved inhibitors, targeting EV-D68 3C protease offers a promising avenue for antiviral drug discovery^17^.

There is no specific treatment for Entrovirus-D68 while intensive care required for respiratory distress or AFM^18^. Prevention may include good hygiene, avoid crowded areas, surface cleaning. For patients with acute respiratory symptoms ventilation needed. For AFM rehabilitation needed for lost motor function. Further research is ongoing for antiviral drugs targeting the polymerase and proteases of virus, but no specific drug has yet been approved^19^. By targeting the polymerases which replicate the viral genome and protease which split the polyprotein into functional viral protein could provide potential therapies. Some phytochemicals like polyphenol, alkaloids and polyphenols show some hints in invitro studies for inhibiting viral replication and have some potential for further future development of drugs^20,21^. In this study, we employed an integrated computational approach, including molecular docking, pharmacokinetic predictions, molecular dynamics simulations, and MMGBSA free energy calculations, to identify natural inhibitors with strong binding affinity and stable interactions within the 3C protease active site^22–26^.

## Material and methods

### Data retrieval and preparation

**Fig. 1:**
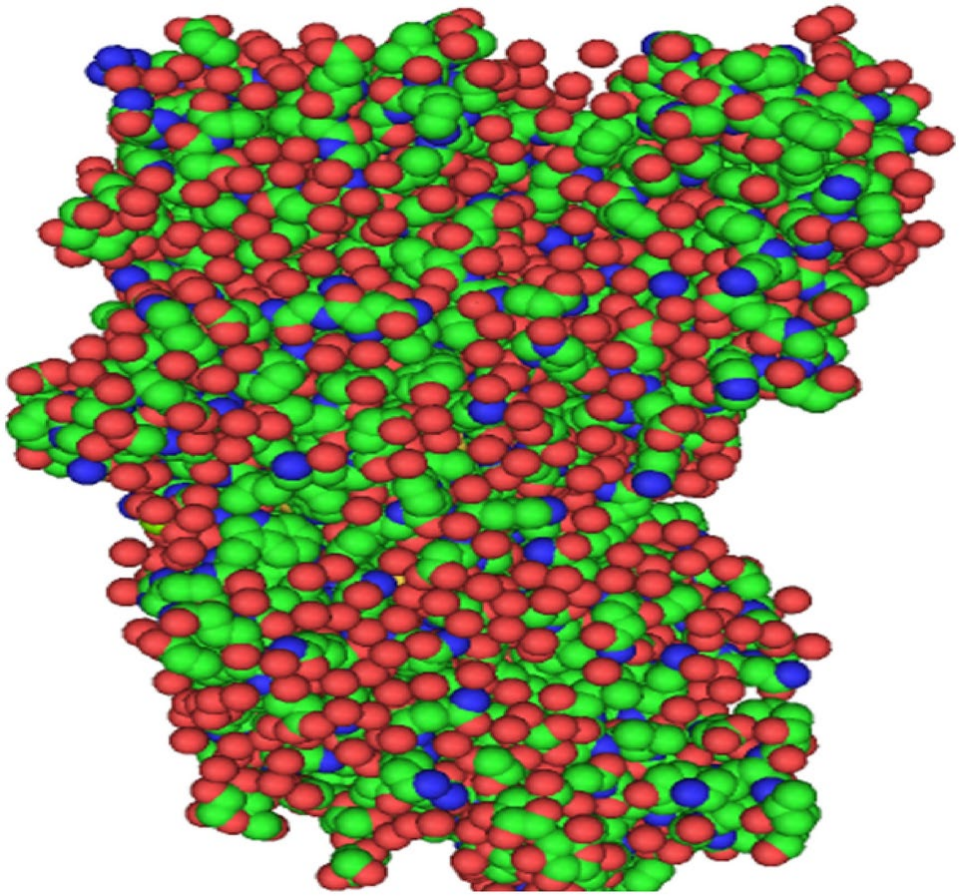
Structure of Enterovirus D68 virus 3C protease (PDB ID: 5GQY) with water molecules, hetero atoms and co-crystallized ligands.

Enterovirus D68’s 3C protease’s 3D configuration was obtained from the Protein Data Bank^27^ with PDB ID 5QGY (Fig. 1). Using an X-ray diffraction method with a resolution value of 2.00 Å, the atomic and molecular structure of the crystal was ascertained. The protein’s A chain was processed for more research to exclude co-crystallized ligands, ions, other chains, the H-atom, and solvent from the protein’s crystal structure. Software called PyMol^28^ was used for setting up the protein structure. The PubChem database^29^ provided the 3D structure of the naturally occurring physiologically active chemicals in SDF format.

### Pharmacokinetics prediction

Pharmacokinetic studies often gather essential data on medication disposal and absorption and demonstrate the relationship between dosage and dosage type. Pharmacokinetic parameters support and define the effectiveness and integrity of drugs in the early stages of CAD^21^. The SwissADME^30^ and admetSAR^21^ servers were used in our investigation to verify the ADMET characteristics of the compounds we were interested in.

### Toxicity prediction

Compound’s toxicity evaluation is an essential part of the drug discovery process. As novel therapeutic products are being developed, most of the best compounds are dropped due to pre-clinical and clinical toxicity. Anticipating the toxicity of compounds helps to maximize lead compounds and reduce risk failure during medication development. Using the publicly available free servers pkCSM and Data Warrior^31^, dangerous characteristics were evaluated. Toxicological qualities may be swiftly examined using the pkCSM server^32^.

### Molecular docking

Molecular docking is put to use to find out the best attachment arrangements and binding affinity between the chemical and the target molecule. The development of new drugs, the interactions between ligands and proteins, and other biological processes may all be better understood via this method^33^. The target macromolecule (5qgy) and the natural chemicals were docked using the AutoDock Vina program^34^. The binding location was chosen for the creation of receptor grids after research on the interaction between the natural compounds inhibitors and the target protein was completed. PyMol^35^, UCSF Chimaera^36^, and Discovery Studio^37^ all showed different kinds of chemical bonds and ligand-binding residues. The Docking RMSD 0 were chosen for further analysis. The docking results were assessed based on binding affinity, key interactions, and Root Mean Square Deviation (RMSD) values. Only docking poses with RMSD values close to the co-crystallized ligand were selected for further analysis, ensuring structural relevance and consistency.

### Molecular dynamic simulation

An MD simulation was execution to focus on the structural stability of the 5gqy-265237 and 5gqy-64982 complexes under particular physiological circumstances^38^. The process of simulation was conducted in 100 ns using the Desmond unit, which is available at Schrödinger suit. For every intricate TIP3P water model, an orthorhombic in periodic border box has been assigned to guarantee that the systems’ volumes stay constant^39^. Na + and Cl-ions were assigned at random and distributed over the solvated system to sustain a salt concentration of 0.15 M. The OPLS3e force field was used to decrease and relaxing the system^40^. After then, the constant NPT ensemble was finished at 300.0 K and 1 atm of pressure^41^. Before everything else, every complex system was made to rest. The result was produced with 100 picosecond (ps) recording intervals. After that a 200 ns simulation have extended for further confirmation of the best protein ligand interaction.

## Results

### Data retrieval

Target 3C protease’s structure was recovered and prepared by eliminating water molecules and minimizing energy. The SDF format of the natural products ligand’s three-dimensional structure was obtained from the PubChem database. Because they provide several advantages against illnesses, natural goods or cures are increasingly being used to treat a variety of inflammatory, diabetic, renal, and cardiac conditions^42,43^. Figure 1 illustrates the structure of the Enterovirus D68 virus 3C protease (PDB ID: 5GQY), showcasing the spatial arrangement of water molecules, heteroatoms, and co-crystallized ligands.

### Pharmacokinetics analysis of natural products

The study of ADME properties that dictate the potency and duration of the effects of a medicinal product is typically referred to as pharmacokinetics. Among all the observed natural compounds, pharmacokinetics assessment is a preliminary step that aids in identifying an effective medication molecule^36^. The features under investigation include MW, GI absorption, HB-donors, HB-acceptors, rotatory bonds, BBB penetration, Log S (ESOL), Consensus Log Po/w, and drug-likeliness qualities of Lipinski rules. For every chemical, we adhered to Lipinski’s rule of five. For additional examination, compounds that satisfied each of these five criteria were selected. Due to their noncompliance with the regulations, several substances were excluded^44^. The pharmacokinetic profiles of substances CID: 265,237 and CID: 64,982 were meticulously assessed, with the results summarized in Table 1. Their favorable pharmacokinetic attributes suggest a high likelihood of success in clinical trials, minimizing the risk of trial failure and underscoring their therapeutic potential.

**Table 1:** The pharmacokinetics properties of the top five selected compounds.

Features	Berberine CID: 2353	Baicalin CID: 64,982	Emodin CID: 3220	Epigallocatechin Gallate CID: 65,064	Withaferin A CID: 265,237
Mol_Weight	336.367	446.4	270.24	458.375	470.606
Rotatable bonds	2	10	0	3	3
H-Bond Acceptors	4	4	5	11	6
H-Bond Donors	0	6	3	8	2
Intestinal absorption (human)	97.147	26.224	74.485	47.395	85.345
Blood Brain Barrier permeability	0.198	-1.331	-0.727	-2.184	-0.03
LOGP	3.0963	0.1422	1.88722	2.2332	3.3529
Caco2 permeability	1.734	-0.67	-2.737	-1.521	0.829

### Toxicity evaluation

A preliminary evaluation of the compound’s toxicology is essential in the field of medication research and development. Toxicological endpoints including mutational capacity, carcinogenicity, and other traits can be investigated both statistically and independently to assess the substance’s toxicity^45,46^. If a substance meets pharmacokinetic and toxicity requirements, it can be accepted orally.

Testing for or analyzing the potential negative effects of chemicals, drugs, or other substances on live organisms is known as toxicity analysis or toxicity testing^47^. Assessing these substances’ safety and potential risks to the environment and human health is the main goal of a toxicological investigation. Occasionally, the molecules created as potential drugs can be toxic and harmful to another organ, resulting in organ toxicity in both human and animal bodies^48^. Therefore, a control was used in this investigation to evaluate the toxicity of the three lead compounds**.** Table 2 shows the top five of the selected compounds (including top two, CID: 265,237 and CID: 64,982) have reasonable toxicity characteristics.

**Table 2:** The Toxicity properties of top five compounds.

Features	Berberine CID_2353	Baicalin CID: 64,982	Emodin CID: 3220	Epigallocatechin Gallate CID_65064	Withaferin A CID_265237
Hepatotoxicity	Yes	No	No	No	No
Ames toxicity	Yes	NO	No	No	No
Oral Rat Acute Toxicity	2.571	2.634	2.116	2.522	2.779
Oral Rat Chronic Toxicity	1.89	4.536	2.074	3.065	0.918
Skin Sensitization	NO	NO	NO	NO	NO

### Molecular docking analysis

The molecular docking research was carried out to comprehend to check the molecular interactions and binding affinities between the drugs natural products being studied and the target 3C protease^49^. The top two compounds with the most exceptional docking scores were chosen for additional analysis. Molecular docking was performed on the natural drug-like compounds that had no violations. With impressive docking scores of -10.7 kcal/mol and -9.5 kcal/mol, CID: 265,237 and CID: 64,982 emerged as the top-performing compounds due to their superior binding affinities, as detailed in Table 3**.** This table provides a comprehensive comparison of all tested compounds, clearly highlighting the exceptional binding interactions of these two candidates with the target biomolecules.

**Table 3:** Chemical Profiles, 3D Structures, and Binding Affinities of the Top Five Compounds.

**.**PubChem CID	Compound name	3D structure	Docking score (Kcal/mol)
CID_265237	Withaferin-A	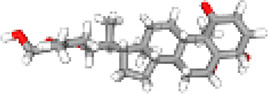	-10.7
CID_64982	Baicalin	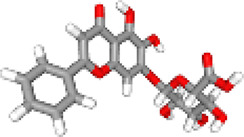	-9.5
CID_3220	Emodin	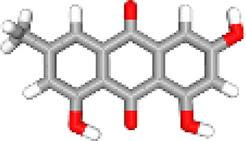	-9.3
CID_ 65,064	Epigallocatechin Gallate	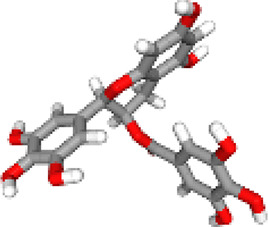	-9.2
CID_2353	Berberine	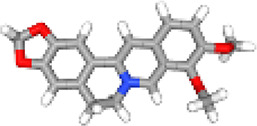	-9

The redocking results with the co-crystallized ligand confirm the reliability of our molecular docking approach. The superimposition of the redocked pose onto the original co-crystallized ligand demonstrates a close alignment, indicating accurate pose prediction. The Root Mean Square Deviation (RMSD) between the docked and crystallographic ligand is within an acceptable range 0.272 Å (< 2.0 Å), suggesting that AutoDock Vina effectively reproduced the experimentally observed binding conformation (Figure S1). Key interactions, including hydrogen bonds and hydrophobic contacts, remain consistent with the native binding mode, further validating the docking protocol. These results confirm the robustness of the docking methodology and provide confidence in the predicted binding orientations of the screened natural compounds.

### Interactions analysis of proteins and ligands

The molecular interactions between withaferin-A and Baicalin ligands and 3C protease receptor were visualized using PyMol, resulting in the formation of many interactions such as hydrogen bonds. The PyMol program was used to visualize the two withaferin-A and Baicalin compounds that passed the pharmacokinetics as well as toxicity analysis and shown the greatest binding affinity throughout the virtual screening phase to determine the interacting residues^49^.

**Fig. 2:**
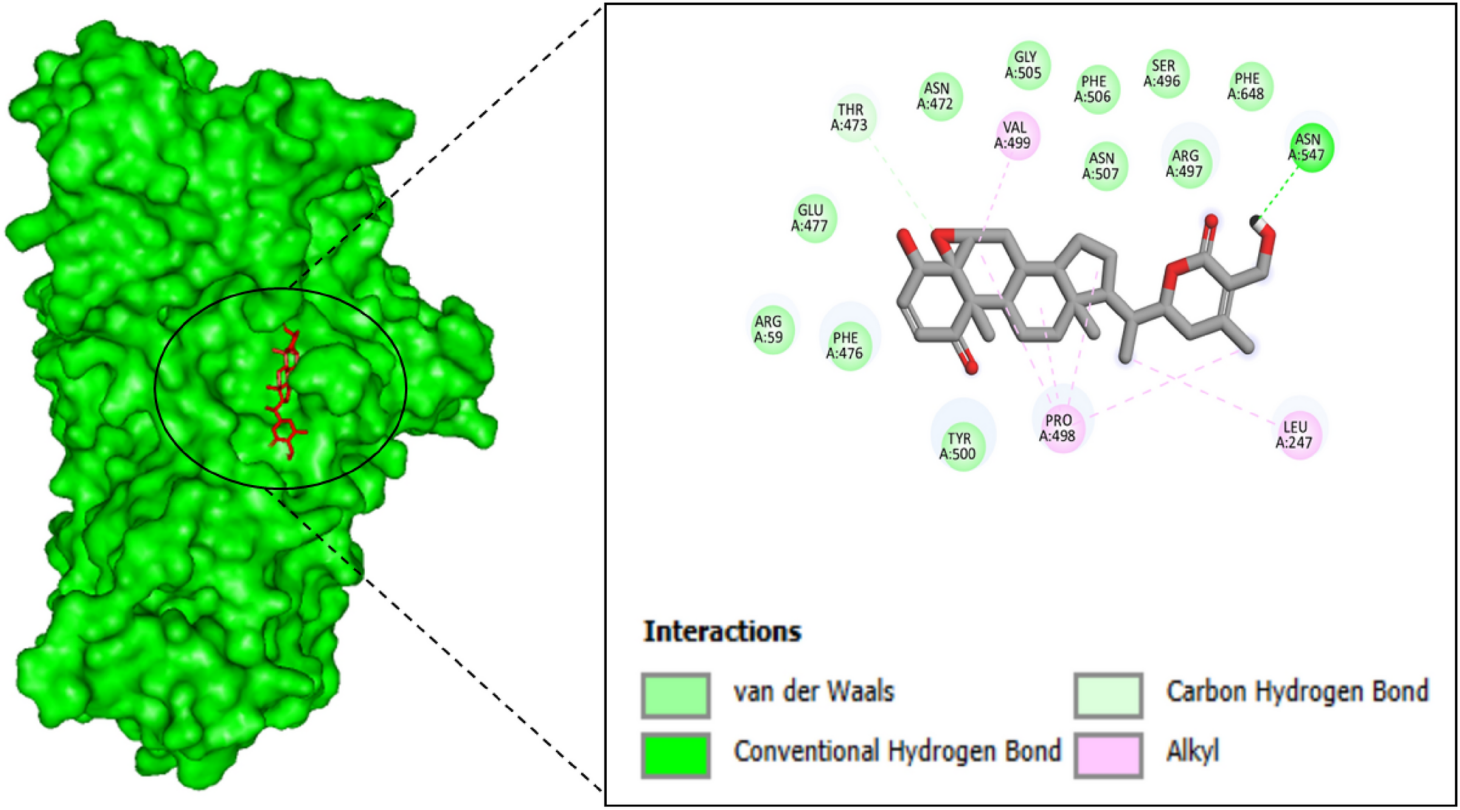
The interactive residues of 3C protease receptor with Withaferin-A. The binding interactions between the 3C protease receptor (represented by the green surface model on the left) and the compound Withaferin-A (depicted in red sticks). The left panel shows the surface structure of the 3C protease, with the binding site highlighted in an oval where the interaction with Withaferin-A occurs. The right panel zooms in on the molecular details of the interaction between Withaferin-A and key residues of the 3C protease receptor. The figure highlights several types of interactions, such as van der Waals forces (shown in green), conventional hydrogen bonds (shown in light green), and carbon-hydrogen bonds (in pink). Important residues of the protease receptor involved in these interactions include LEU247, ASN547, VAL499, TYR500, and PRO498, among others. The specific residues involved in the binding are labeled, with the interaction types distinguished by color coding in the figure legend. These interactions are crucial for the stability and affinity of Withaferin-A in binding to the 3C protease receptor, which may contribute to its potential as an antiviral agent against EV-D68.

The stability of the ligand within the binding pocket is primarily governed by a combination of hydrogen bonding, hydrophobic interactions, and electrostatic interactions. These interactions are crucial for maintaining the stability and affinity of the ligand to the receptor. The interactive residues of Withaferin-A (CID_265237) compound with receptor are LEU247, THR473, PRO498, VAL499 and ASN547. The interactive residues of Baicalin (CID_64982) with receptor are VAL97, THR99, LEU238, ARG241, ARG242, ASP424 and HIS427. Hydrogen bonds, which were observed between key residues and the ligand, play a central role in anchoring the ligand within the binding site. Withaferin-A forms stable hydrogen bonds with residues like ASN547, contributing to its strong binding affinity (Fig. 2). Similarly, Baicalin engages in hydrogen bonding with ASP424 and HIS427, which helps stabilize the complex.

**Fig. 3:**
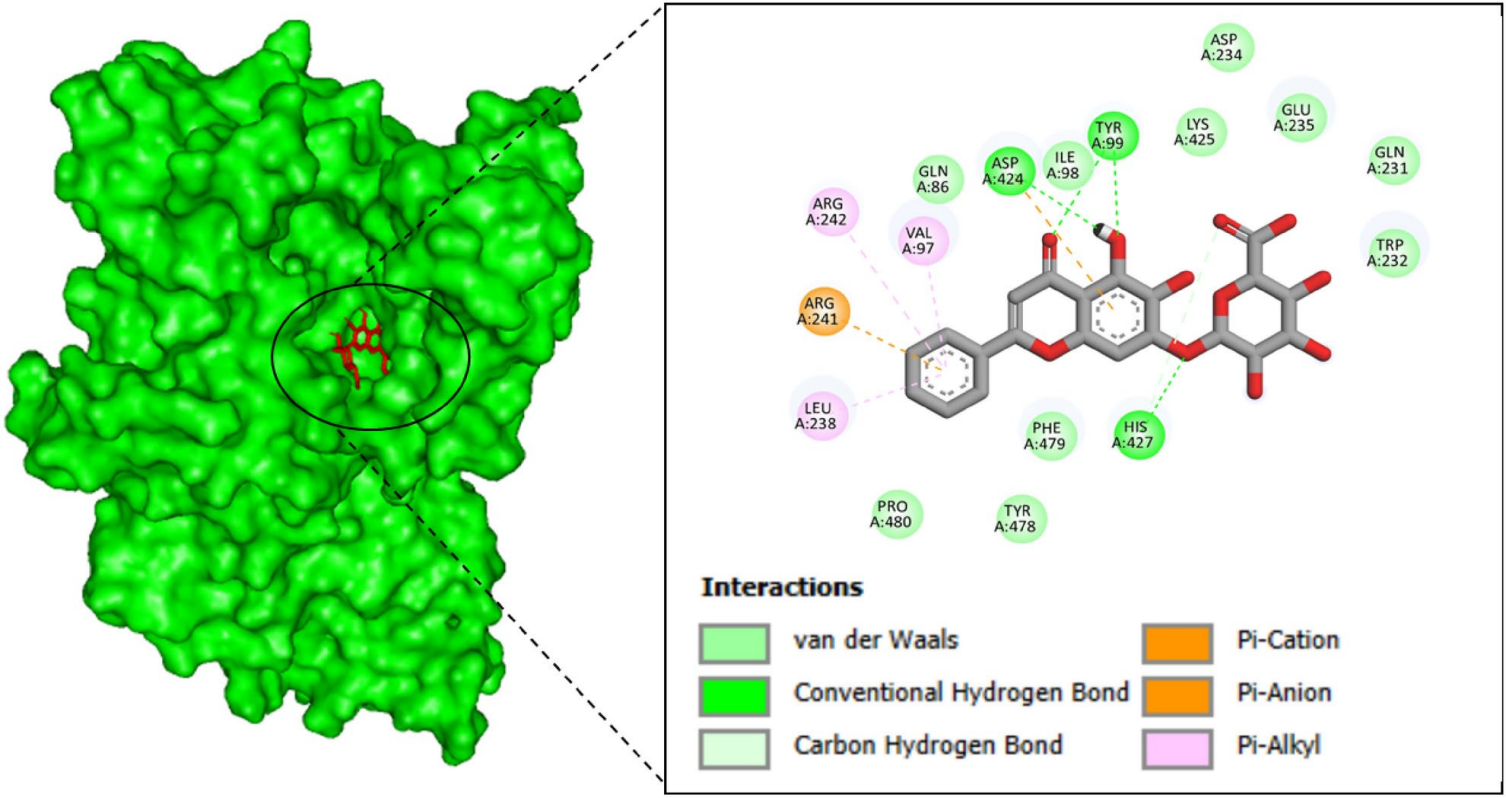
The interactive residues of 3C protease receptor with Baicalin. This figure illustrates the binding interactions between the 3C protease receptor (represented by the green surface model on the left) and the compound Baicalin (depicted in red sticks). The left panel highlights the surface structure of the 3C protease, with the binding site enclosed in an oval to show where Baicalin interacts with the receptor. The right panel zooms in to reveal the detailed molecular interactions between Baicalin and the 3C protease receptor, focusing on key residues involved in binding. The interactions include van der Waals (green), conventional hydrogen bonds (light green), carbon-hydrogen bonds (pink), Pi-Cation interactions (orange), and Pi-Anion interactions (purple). Key residues of the protease receptor involved in these interactions include TYR99, ASP424, GLU425, PHE479, ARG241, and PRO480, among others. The color-coded interactions indicate the strength and nature of the bonding, with the Pi-Cation and Pi-Anion interactions suggesting electrostatic attractions between the aromatic rings of Baicalin and the charged residues of the protease. These interactions play a crucial role in stabilizing the binding of Baicalin to the 3C protease receptor, further supporting its potential as an antiviral agent.

In addition to hydrogen bonding, hydrophobic interactions between non-polar residues, such as VAL499 and LEU247, contribute significantly to the overall binding strength, facilitating the proper alignment of the ligand within the binding pocket. Electrostatic interactions, including Pi-Cation and Pi-Anion interactions, also play a critical role in stabilizing the ligand-receptor complex. Baicalin forms Pi-Cation interactions with ARG241 and ARG242, and Pi-Anion interactions with PHE479 and HIS427, further stabilizing the binding of the ligand within the receptor’s pocket (Fig. 3). These interactions, including both polar and non-polar forces, are essential for the stability and effective binding of Withaferin-A and Baicalin to the 3C protease receptor, supporting their potential as effective antiviral agents.

### Molecular dynamics simulation

**Fig. 4:**
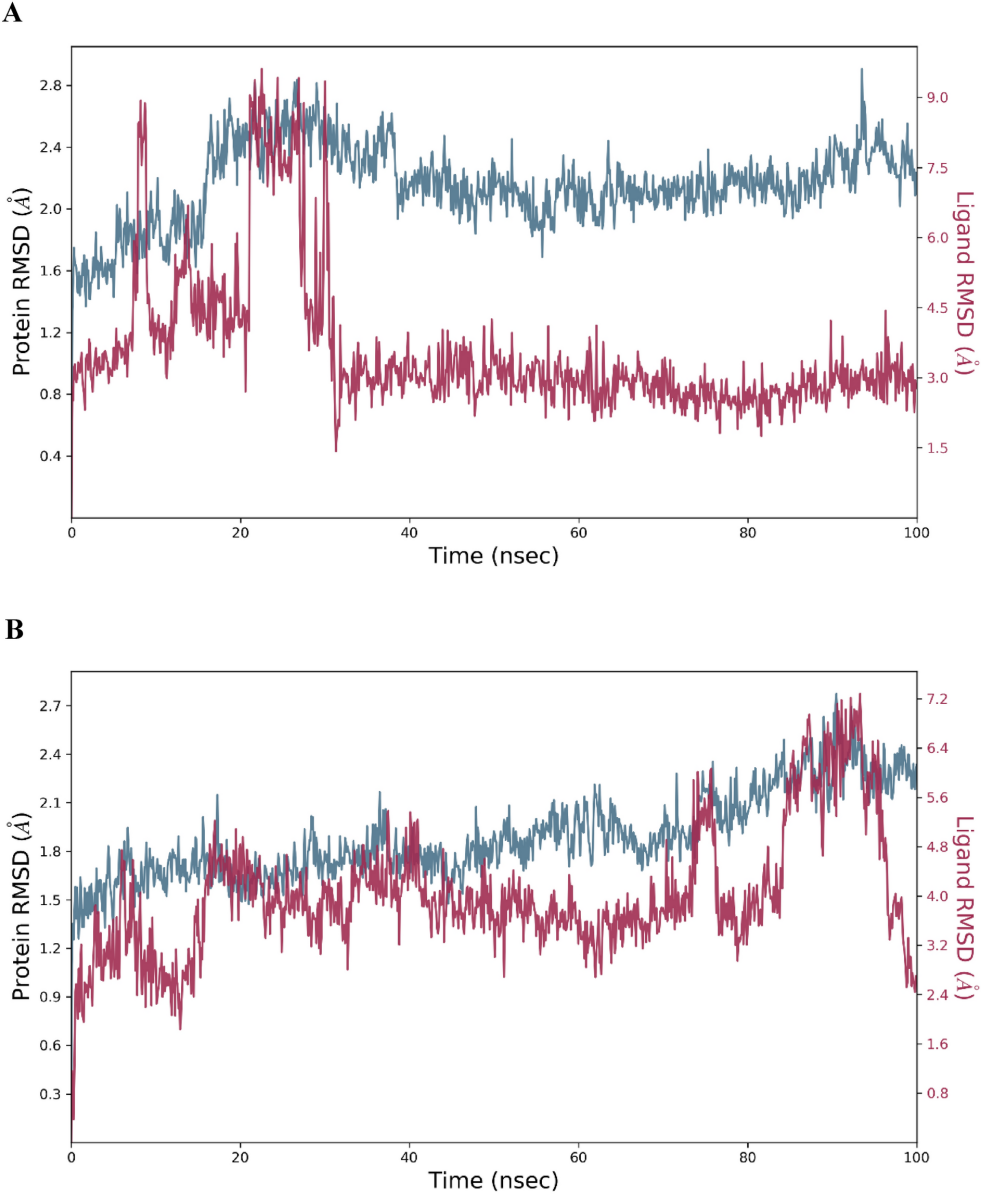
RMSD for the atoms of C-alpha. The change in protein RMSD in gray color over time was shown on the Y-axis. Additionally, the ligand RMSD fluctuation in red color over time was shown on the Y-axis.

The protein in the complex 3C protease and Withaferin-A (CID: 265,237) attained the stability level at 25 ns, based on the RMSD plot **(**Fig. 4A**).** For the rest of the simulation, RMSD value variations stay within 1.5 Angstrom. The ligand Withaferin-A (CID: 265,237) fit to protein RMSD values varied within 1.5 Angstrom after attaining equilibrium and stayed stable for the length of the simulation up to 100 ns. RMSD results indicated that the proteins in the complex 3C protease and (CID_64982) Baicalin (Fig. 4B) stabilized at 20 ns. Following this, throughout the duration of the simulation, RMSD value variations stay within 1.5 Angstrom^49^. When the ligand (CID_64982) Baicalin fit to protein reached equilibrium, the RMSD values ranged between 2.5 and 3.0 Angstrom and were constant throughout the simulation, with the exception of a little increase at 90 ns **(**Fig. 4B**).**

**Fig. 5:**
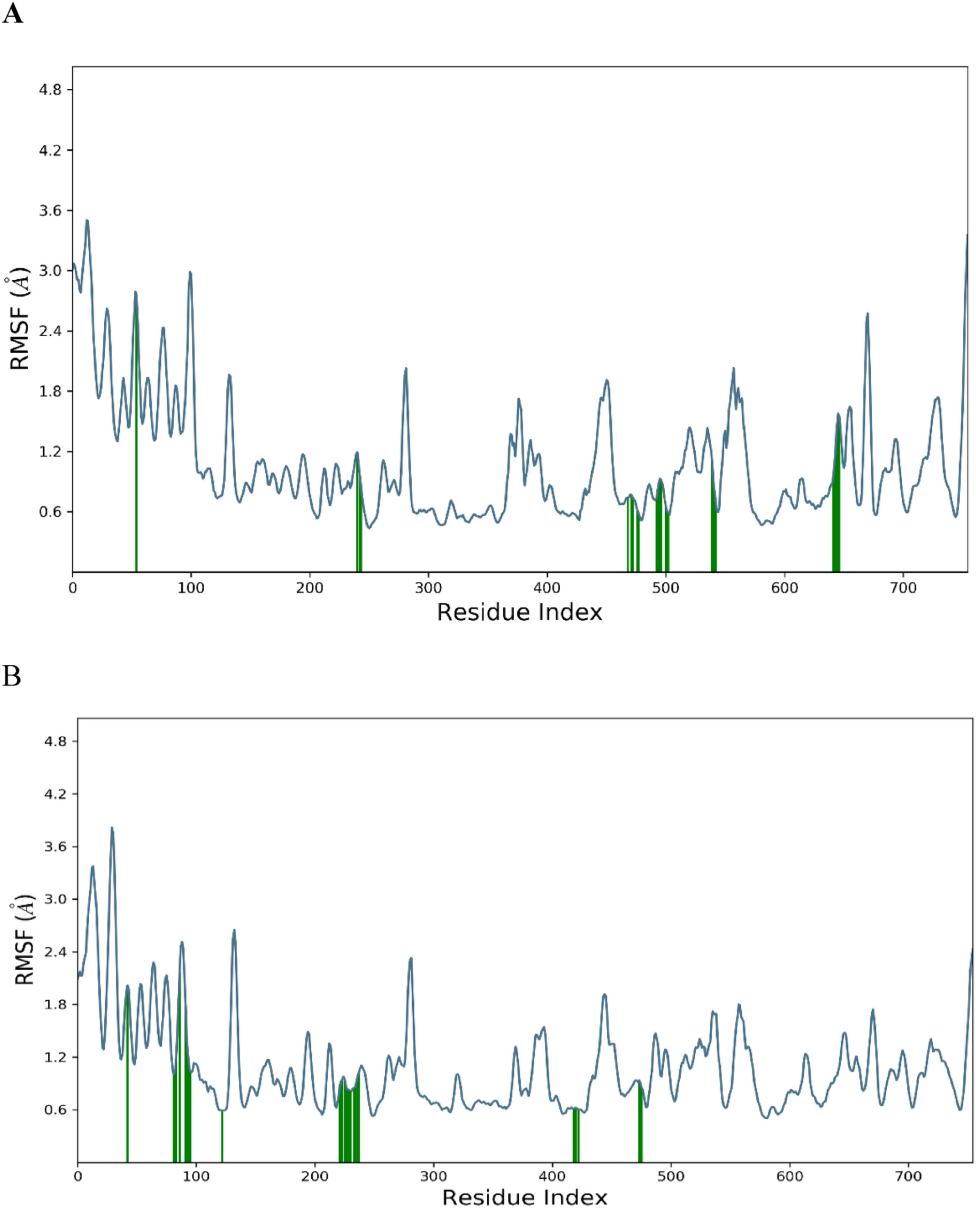
RMSF of the 3C protease receptor docked complex (**A**) Withaferin-A and (**B**) Baicalin.

The 3C protease protein–ligand-coupled RMSF value **(**Figs. 5A and 5B**).** According to simulated trajectories, the residues with greater peaks are found in loop regions or N and C-terminal zones. Low RMSF values of binding site residues indicate the stability of ligands (CID: 265,237 and CID: 64,982) binding to the protein.

**Fig. 6:**
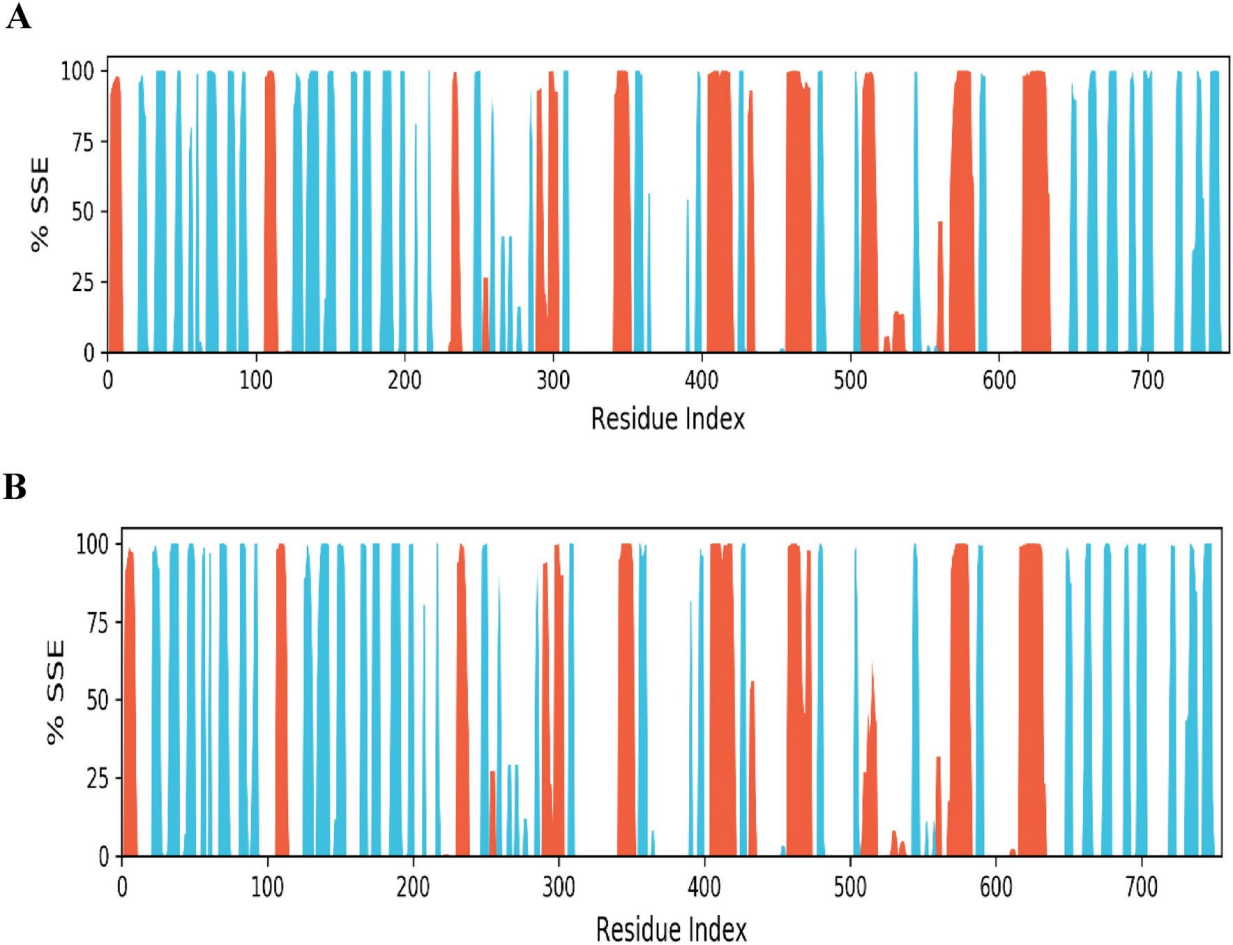
Histogram 3C protease structural complex of protein–ligand interface (**A**) According to simulated trajectories, the residues with greater peaks are found in loop regions or N and C-terminal zones. Low RMSF values of binding 1200 site residues indicate the stability of ligands (CID: 265,237 and CID: 64,982) binding to the protein.

Overall secondary structural elements were found to be 41.52 percent, with helix and strand percentages in protein–ligand complex 3C protease and Withaferin-A (CID: 265,237) being 17.21 and 24.21 percent, respectively **(**Fig. 6A**).** Overall secondary structural elements were discovered to be 39.83 percent, with the percentages of helix and strand in protein–ligand complex 3C protease and (CID_64982) being 15.89 percent and 23.94 percent, respectively **(**Fig. 6B**).**

The chosen compounds, Baicalin (CID_64982) and Withaferin-A (CID: 265,237), had free binding energies of -68.18223059 kcal/mol and -77.98376587 kcal/mol, respectively (Table 4*).*

**Table 4:** MM-GBSA Analysis of 3C Protease: Energetic Contributions of Complexed Ligands Withaferin-A and (B) Baicalin.

Energies (Kcal/mol)	Withaferin-A (CID: 265,237)	Baicalin (CID_64982)
dG_bind_	-77.98376587	-68.18223059
dG_b_Lipo	-51.51114073	-31.80787647
dG_b_vdW	-43.18354069	-49.55308155
dG_b_H_bond_	-0.951544452	-3.4850661
dG_b_Coulomb	-8.044983856	-31.28288014

**Fig. 7:**
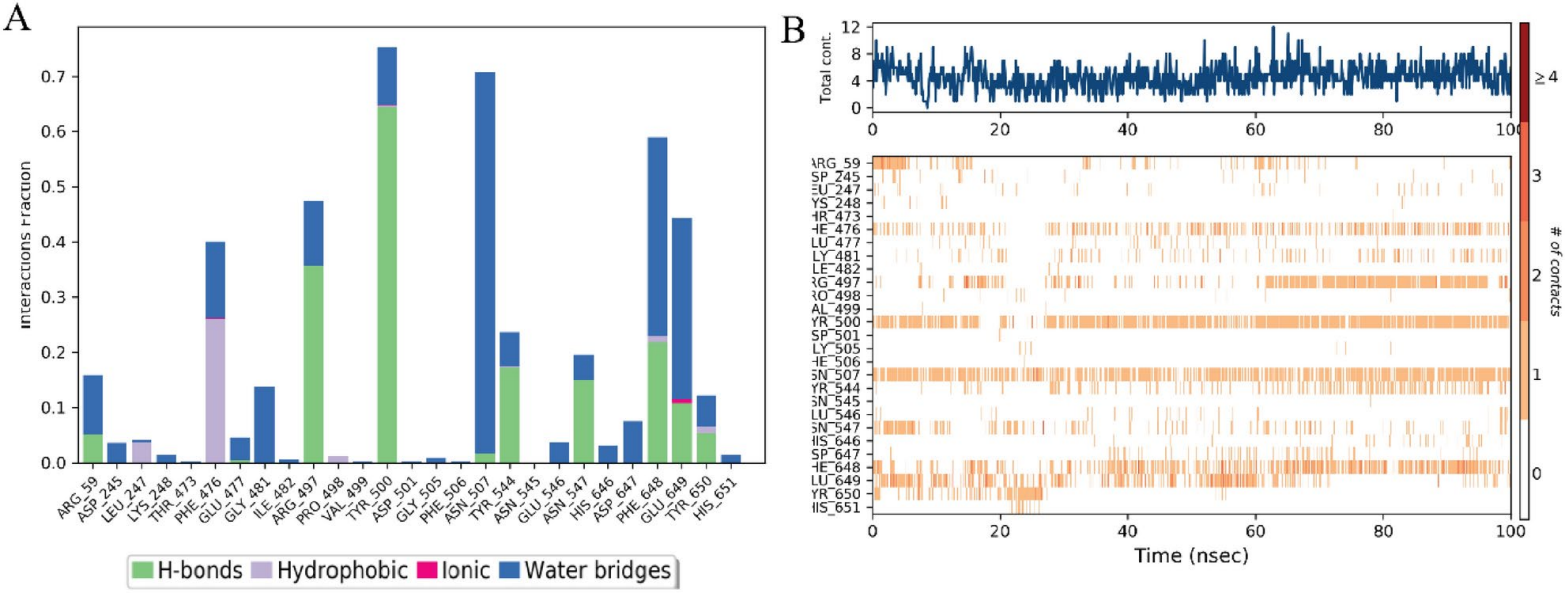
Histogram 3C protease structural complex of protein–ligand interface (**A**) The stacked bar chart represents the interaction fractions of different residue contacts within the Withaferin-A (CID: 265,237) complex over a 100 ns simulation. (**B**) The contact timeline illustrates the variation in protein–ligand contacts over the 100 ns simulation. The heatmap intensity represents the frequency of interactions at each simulation frame, highlighting the persistence of hydrogen bonding and water bridge interactions.

The protein–ligand (PL) contact analysis of the Withaferin-A (CID: 265,237) complex over a 100 ns molecular dynamics (MD) simulation provides insights into the stability and nature of interactions. The hydrogen bonding and water bridge interactions were found to be the most persistent, playing a crucial role in stabilizing the ligand within the binding pocket throughout the simulation. Residues such as ARG-59, TYR-500, and GLU-649 frequently participated in hydrogen bonding, indicating their significant role in ligand stabilization. Hydrophobic interactions, primarily involving LEU-247 and PHE-506, appeared intermittently, suggesting that non-polar forces contribute to ligand binding but are less stable over time. Ionic, Pi-cation, and Pi-Pi interactions were relatively rare, implying that electrostatic and aromatic stacking forces had a minor role in maintaining the ligand–protein complex (Fig. 7).

The protein–ligand (PL) contact analysis of the Baicalin (CID: 64,982) complex over a 100 ns molecular dynamics (MD) simulation highlights key interaction trends. Hydrogen bonds and water bridges were the most frequent and persistent interaction types, indicating that polar interactions play a crucial role in stabilizing the ligand within the binding pocket. Key residues such as GLU-235 and ASP-424 were frequently involved in hydrogen bonding, suggesting their importance in ligand stabilization.

**Fig. 8:**
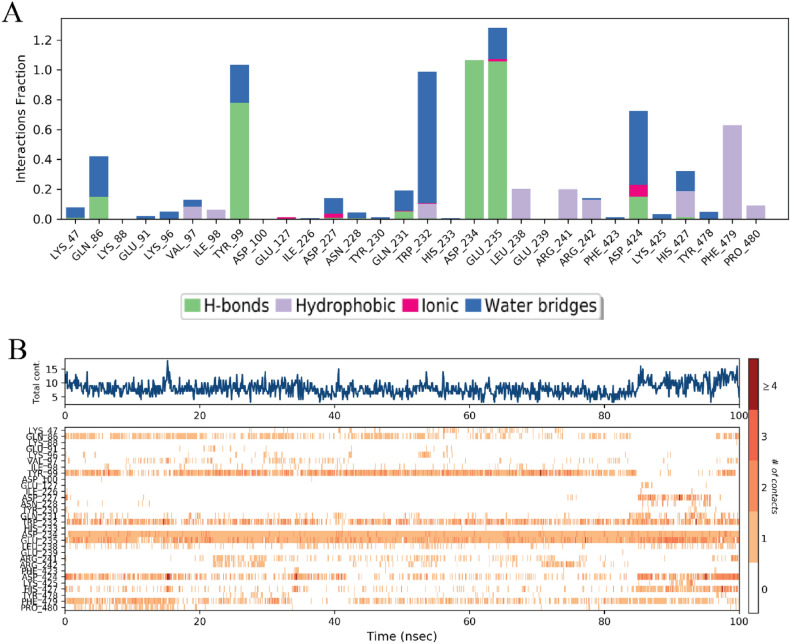
Histogram 3C protease structural complex of protein–ligand interface (**A**) Residue-wise Interaction Fractions: The stacked bar chart represents the fraction of different interaction types observed between Baicalin (CID: 64,982) and the protein over the 100 ns simulation. Interaction types include H-bonds (green), hydrophobic interactions (orange), ionic (blue), metal (yellow), Pi-cation (purple), Pi-Pi stacking (brown), and water bridges (gray). (**B**) Interaction Contacts Over Time: The timeline plot shows the evolution of protein–ligand interactions throughout the simulation. The intensity of the heatmap represents the frequency of interactions, with hydrogen bonds and water bridges dominating, while hydrophobic and aromatic interactions fluctuate over time.

Hydrophobic interactions were observed intermittently, particularly with residues contributing to non-polar stabilization. Pi-Pi and Pi-cation interactions were present but occurred less frequently, indicating that aromatic stacking and electrostatic contributions were minimal. Ionic and metal interactions were almost absent, suggesting that charge-based interactions do not significantly contribute to the ligand’s binding stability (Fig. 8).

### Re-Simulation result

**Fig. 9:**
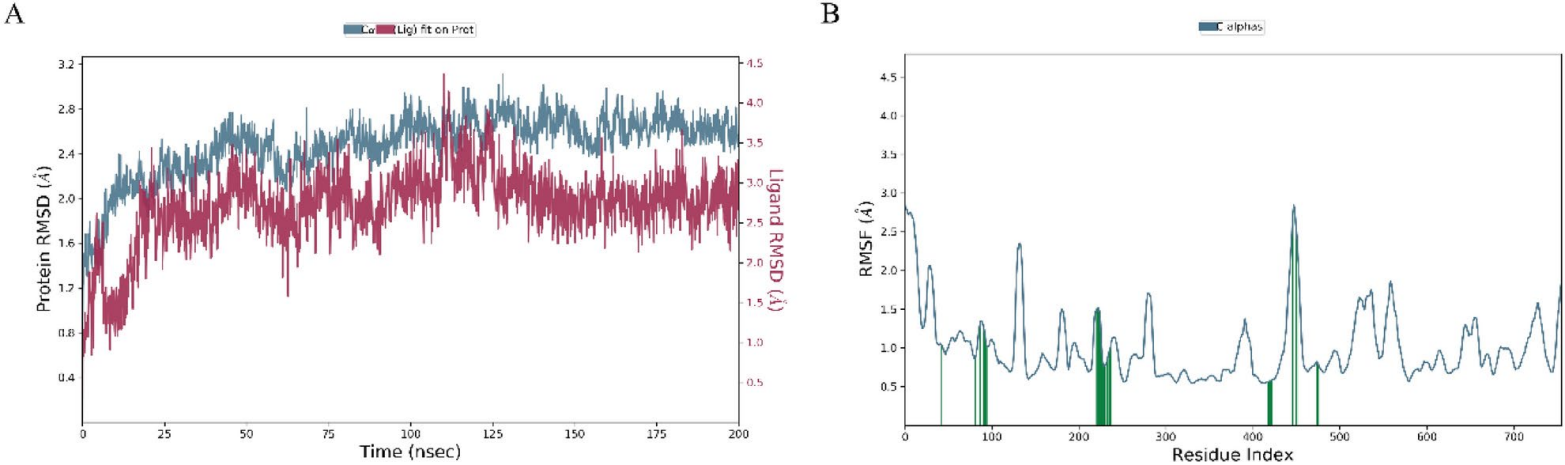
A 200 ns simulation analysis of Baicalin (CID: 64,982) with protein. (**A**) Protein and Ligand RMSD: The Root Mean Square Deviation (RMSD) plot shows the structural stability of the protein (blue, left y-axis) and ligand (red, right y-axis) over 200 ns. The protein RMSD stabilizes around 2.5–3.0 Å, indicating a relatively stable conformation, while the ligand RMSD fluctuates between 1.5–3.5 Å, suggesting moderate movement within the binding site. (**B**) Protein Residue Flexibility (RMSF): The Root Mean Square Fluctuation (RMSF) plot represents residue-wise flexibility, with peaks exceeding 2.5 Å at certain residues (~ 100, ~ 200, ~ 450), indicating flexible loop regions, while residues with RMSF < 1.0 Å correspond to the stable core.

The RMSD and RMSF analysis of the Baicalin (CID: 64,982) complex over 200 ns reveal the stability and flexibility of the system. In panel A, the protein RMSD stabilizes around 2.5–3.0 Å after ~ 50 ns, indicating structural stability, while the ligand RMSD fluctuates between 1.5–3.5 Å, suggesting moderate flexibility within the binding pocket. In panel B, the RMSF values range from 0.5 to 3.0 Å, with peaks exceeding 2.5 Å at certain residues (~ 100, ~ 200, ~ 450), likely indicating flexible loop regions. Residues with RMSF < 1.0 Å suggest a stable core structure. These values indicate that Baicalin remains bound but exhibits dynamic movement within the binding site, which may influence its binding affinity and interaction stability (Fig. 9.

## Discussion

Enterovirus D68 causes respiratory infections that resemble the common cold. It may sometimes result in serious respiratory problems and paralysis-causing neurological illness outbreaks^45^. While most patients recover some muscular function, some people may have long-term health problems. Enterovirus D68 becomes to be widespread. In fact, the majority of adults over five exhibit signs of a prior illness. Although neurological issues are comparatively uncommon, catastrophic EV-D68 epidemics have occurred^50^. Creating effective medications to combat and stop future epidemics of the Enterovirus D68 is crucial as well. We utilized the natural products as therapeutics to treat the Enterovirus D68 by targeting the 3C protease receptor^40^.

**Fig. 10:**
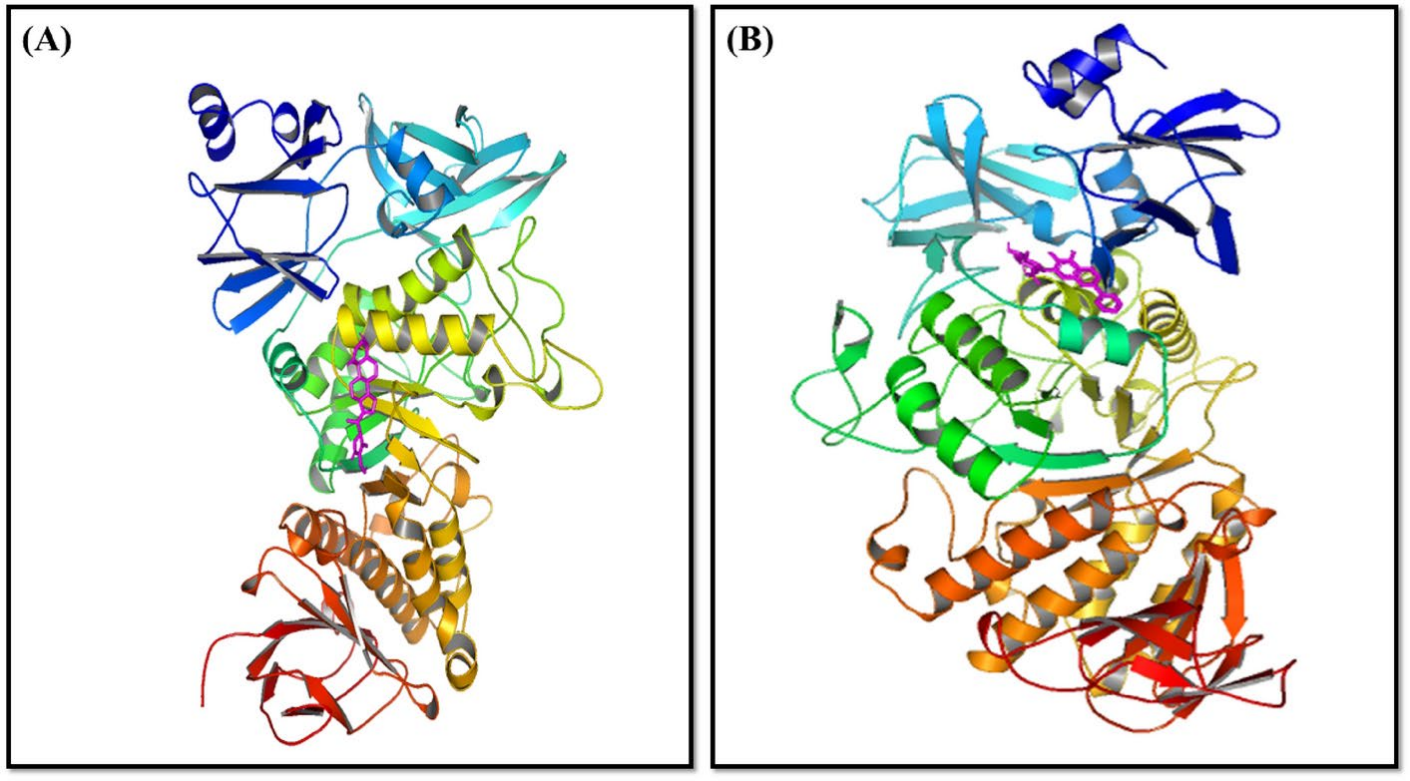
Structures of the Top Two Complexes Showing Target Receptor Interactions with Ligands. (**A**) 3C Protease Receptor Bound to Withaferin-A and (**B**) 3C Protease Receptor Bound to Baicalin.

The PDB database was accessed to get the structure of the 3C protease receptor with PDB ID 5GQY. SDF format structures of a few natural product ligands were obtained from the PubChem database. The pharmacokinetic characteristics of a few chosen natural compounds have been examined. The receptor-binding characteristics, ADME toxicity, and drug-likeness of a few natural compounds were further investigated. How a medication travels through the body is determined by ADME properties^40^. The way that a substance passes through the bodies of both humans and animals is greatly influenced by these characteristics. In order to meet the requirements, set by clinical trials and ascertain the potential efficacy of the drug candidate, it is crucial to optimize the pharmacokinetic parameters throughout the drug development process^48^. As per the Lipinski criteria for drug-likeness, a pharmacological agent is deemed appropriate for oral administration if its molecular weight is less than 500 g/mol, its ClogP is less than 5, its HB-accepting atoms are less than 10, and its HB-donating atoms are less than 5. The logP value of a molecule that is thought to be a possible lead chemical indicates its absorption rate. The body’s absorption of medication molecules is inversely connected with the log P value^51^. The LogS number indicates how soluble the potential chemical. “Toxicity” is the term used to describe a chemical’s capacity to damage organs or to endanger people or animals^52^. The ADMET and physicochemical characteristics are predicted using the pkCSM tool. The ADME and toxicity requirements were satisfactorily met by Withaferin-A (CID: 265,237) and Baicalin (CID_64982). Figure 10 illustrates the structures of the top two complexes showing target receptor interactions with ligands: (A) the 3C protease receptor bound to Withaferin-A, and (B) the 3C protease receptor bound to Baicalin.

After preparing the structures of the ligand and the target 3C protease, molecular docking was used to examine the ligand-receptor interaction, binding posture, and binding affinity. The major area of study for screening lead compounds is molecular docking^53^. This is a sophisticated method for identifying the best new chemicals in libraries^35^. Finding the most effective treatment drug to stop Enterovirus D68 infection is the goal of this investigation. Two natural compounds, Withaferin-A (CID: 265,237) and Baicalin (CID_64982), were chosen after molecular docking was performed on selected natural compounds of them. Their binding affinities were -10.7 kcal/mol and -9.5 kcal/mol, respectively.

A molecular simulation enables predictions about the motion of atoms and their interactions over a certain period of time inside a 5gqs receptor or similar molecular structure^54^. MD simulations may be used to ascertain the behavior of cellular molecules at an atomic level. They are indicators of the compounds’ optimal sustainability, and the complexes Withaferin-A (CID: 265,237) and Baicalin (CID_64982) are measured for RMSD. Additionally, the RMSD values of the substances Withaferin-A (CID: 265,237) and Baicalin (CID_64982) showed stability in dynamics respectively. The fact that the residue change is rather small when compared to the native structural elements found in the complex frame demonstrates the stiffness of the receptor structure. The 5gqs receptor’s beginning and finish are said to have the greatest levels of fluctuation because of the existence of the α-helix, β-sheet and terminals (N and C) domains^55^.

To further validate the docking results, a 100 ns molecular dynamics (MD) simulation followed by a 200 ns re-simulation for Baicalin was performed. The RMSD analysis indicated that Withaferin-A stabilized around 1.5 Å after 25 ns, while Baicalin remained stable between 2.5–3.0 Å after 20 ns, with minimal deviations. RMSF analysis highlighted greater flexibility in loop regions, whereas binding site residues exhibited lower fluctuations, confirming stable ligand retention. Protein–ligand (PL) contact analysis further emphasized the dominance of hydrogen bonds and water bridges, reinforcing their role in stabilizing the complexes.

Recent studies have highlighted rupintrivir as a potent inhibitor of the EV-D68 3C protease, with mean 50% effective concentrations (EC₅₀s) ranging from 0.0018 to 0.0030 μM across various EV-D68 strains. Similarly, SG85, a Michael acceptor inhibitor, has demonstrated EC₅₀ values between 0.0022 and 0.0080 μM, indicating strong antiviral activity^56^.

In our study the MMGBSA analysis supported these findings, showing strong binding free energy (ΔGbind) values of -77.98 kcal/mol for Withaferin-A and -68.18 kcal/mol for Baicalin, with van der Waals and lipophilic interactions being the primary contributors. The higher hydrogen bonding contribution (-3.48 kcal/mol) in Baicalin suggests a more extensive polar interaction network, while Withaferin-A exhibits overall stronger affinity. To provide a more comprehensive assessment, future studies could aim to determine the EC₅₀ values of Withaferin-A and Baicalin in standardized assays, facilitating a more direct comparison with known inhibitors like rupintrivir and SG85^56^. These results collectively confirm the stable and favorable binding of both compounds to the EV-D68 3C protease, highlighting their potential as effective antiviral agents, warranting further in vitro and in vivo validation for therapeutic development. The compounds were shown to be active against EV68-3C protease by extensive in-silico investigations.

## Conclusion

In conclusion, this study offers valuable insights into potential therapeutic strategies for combating Enterovirus-D68. Through molecular docking and dynamics simulations, we evaluated the stability and binding affinities of two promising inhibitors, Withaferin-A (CID: 265,237) and Baicalin (CID: 64,982). The identification of specific residues contributing to binding energies provides critical guidance for rational drug design and optimization. The results highlight the potential of these compounds as viable candidates for antiviral therapy, demonstrating their ability to interact effectively with the viral target, 3C protease. The detailed analysis of molecular interactions, combined with the robust stability of these complexes, underscores their therapeutic promise. However, the transition from computational predictions to practical antiviral treatments requires further experimental validation and refinement. Future studies should focus on in vitro and in vivo testing to confirm efficacy, optimize pharmacokinetics, and assess safety profiles. By bridging this gap, the findings of this research could pave the way for the development of innovative, targeted treatments for Enterovirus-D68, addressing a critical need in antiviral therapy.

## Supplementary Information

Supplementary Information 1.

Supplementary Information 2.

## Data Availability

The datasets analysed during the current study is available from the corresponding author on reasonable request.
