# Supplementary figures and images for: Computational discovery of natural inhibitors targeting enterovirus D68 3C protease using molecular docking pharmacokinetics and dynamics simulations
Source: Sci Rep. 2025 Mar 31;15:11015. doi: 10.1038/s41598-025-95163-y (PMC11958634; doi:10.1038/s41598-025-95163-y)

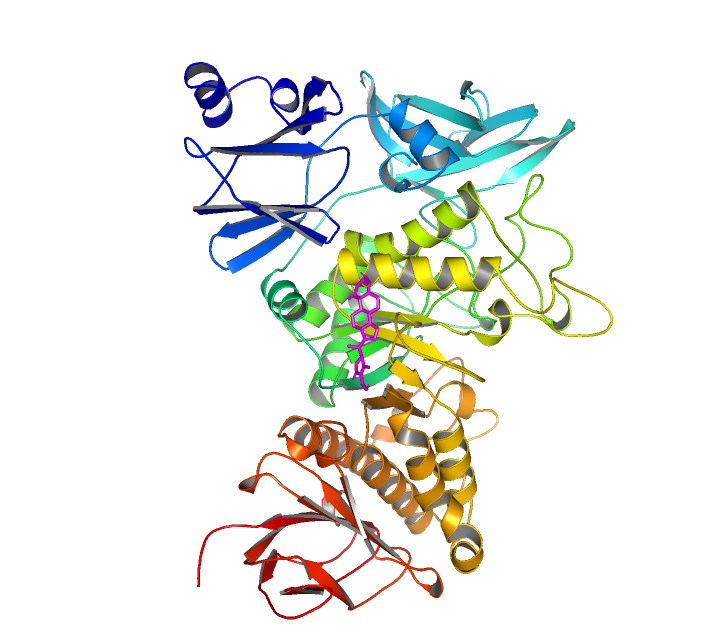

Supplement: Supplementary file 1 — Supplementary Information 1. [file 41598_2025_95163_MOESM1_ESM.zip › docking/37.png]

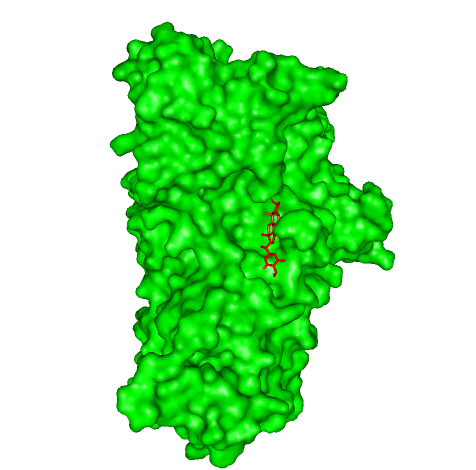

Supplement: Supplementary file 1 — Supplementary Information 1. [file 41598_2025_95163_MOESM1_ESM.zip › docking/5gqy-265237-complex.png]

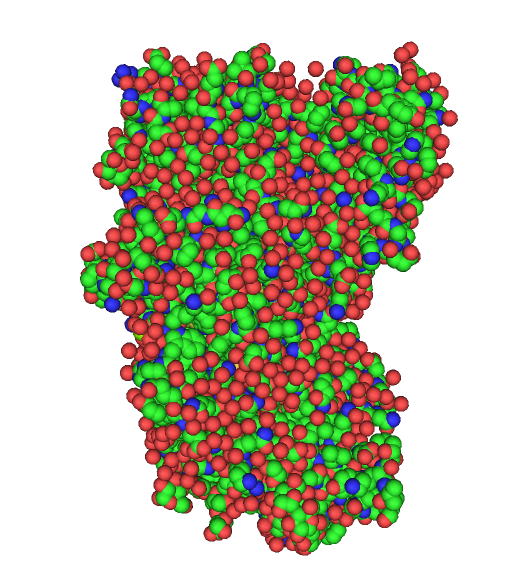

Supplement: Supplementary file 1 — Supplementary Information 1. [file 41598_2025_95163_MOESM1_ESM.zip › docking/5gqy-complex.png]

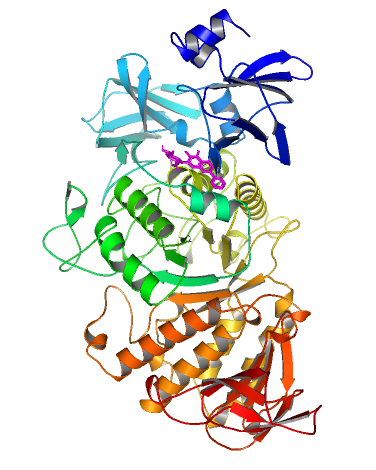

Supplement: Supplementary file 1 — Supplementary Information 1. [file 41598_2025_95163_MOESM1_ESM.zip › docking/82.png]

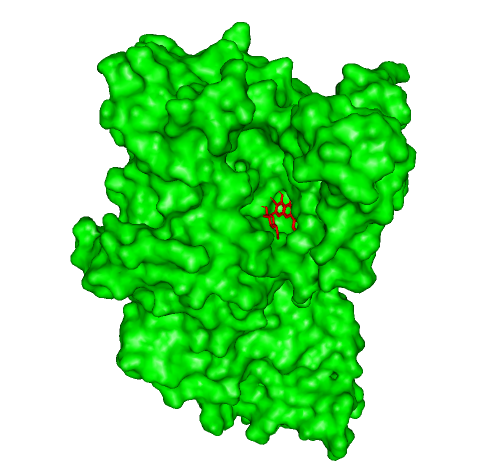

Supplement: Supplementary file 1 — Supplementary Information 1. [file 41598_2025_95163_MOESM1_ESM.zip › docking/82-complexx.png]

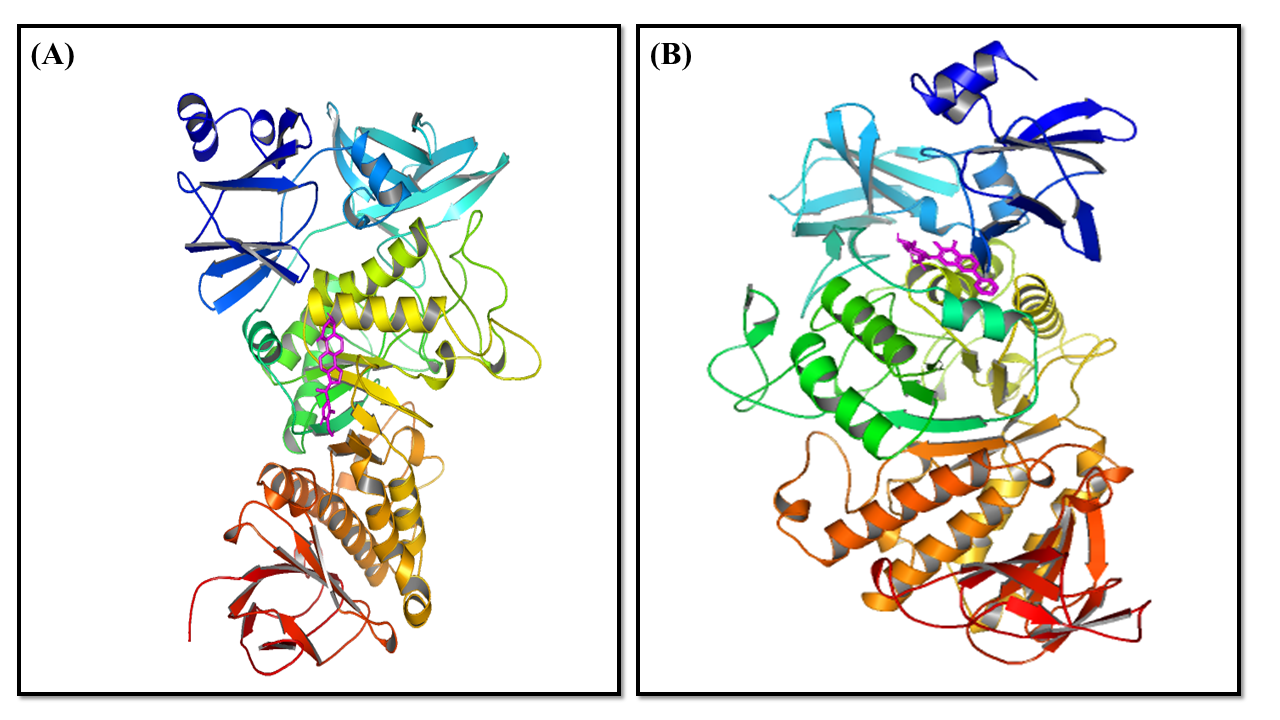

Supplement: Supplementary file 1 — Supplementary Information 1. [file 41598_2025_95163_MOESM1_ESM.zip › docking/Docking complexes.png]

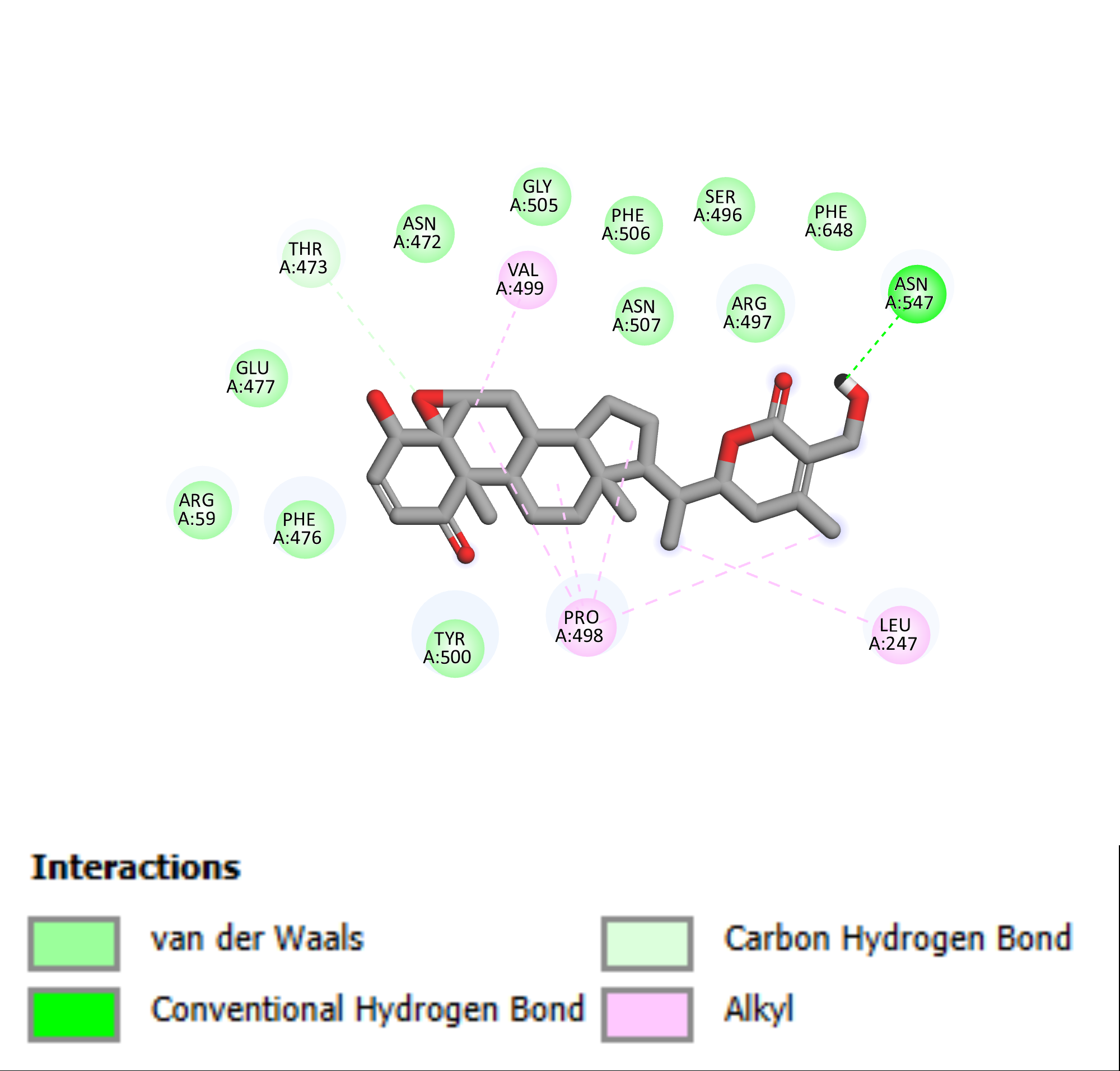

Supplement: Supplementary file 1 — Supplementary Information 1. [file 41598_2025_95163_MOESM1_ESM.zip › docking/Top-2/37.png]

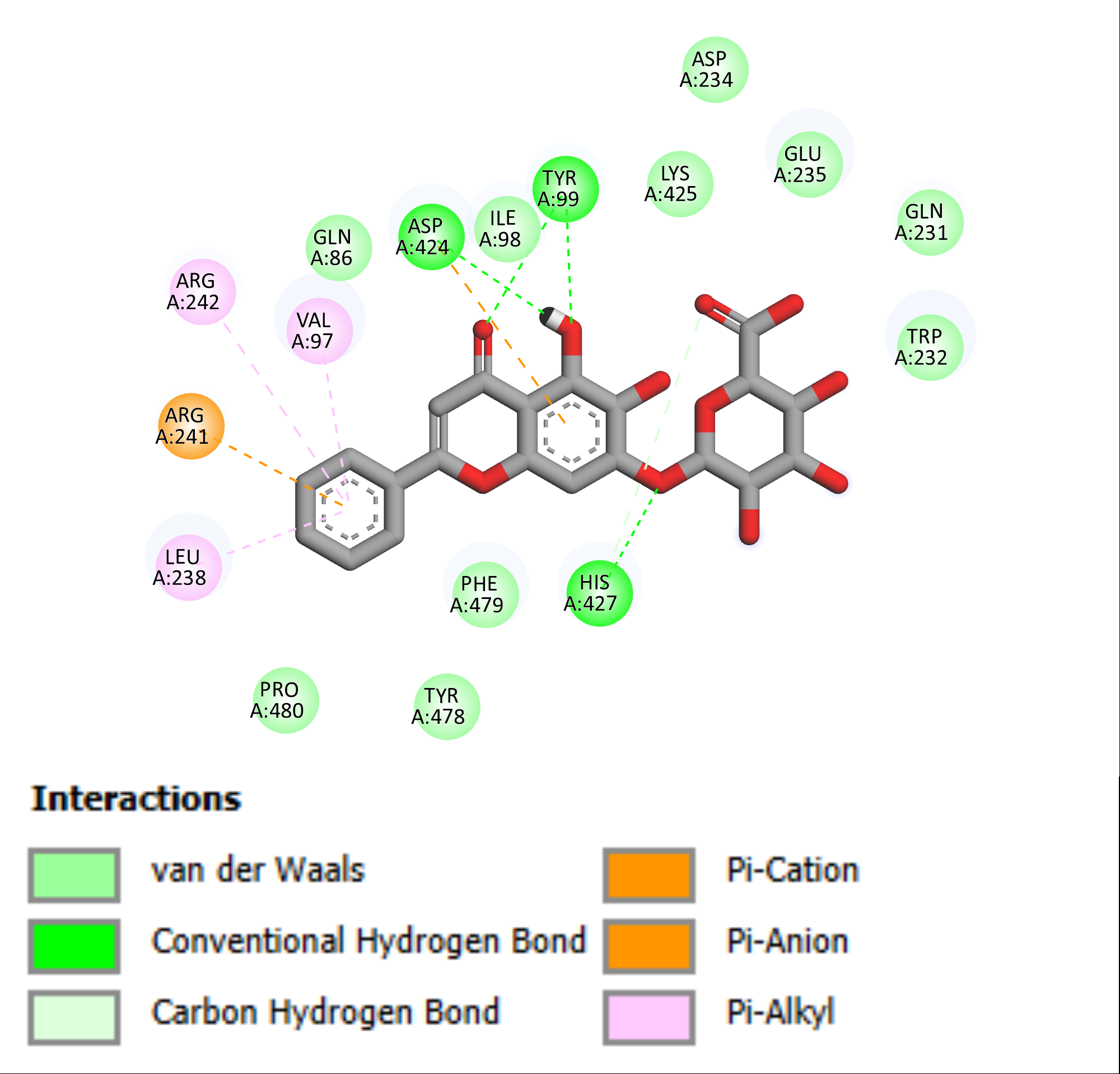

Supplement: Supplementary file 1 — Supplementary Information 1. [file 41598_2025_95163_MOESM1_ESM.zip › docking/Top-2/82.png]

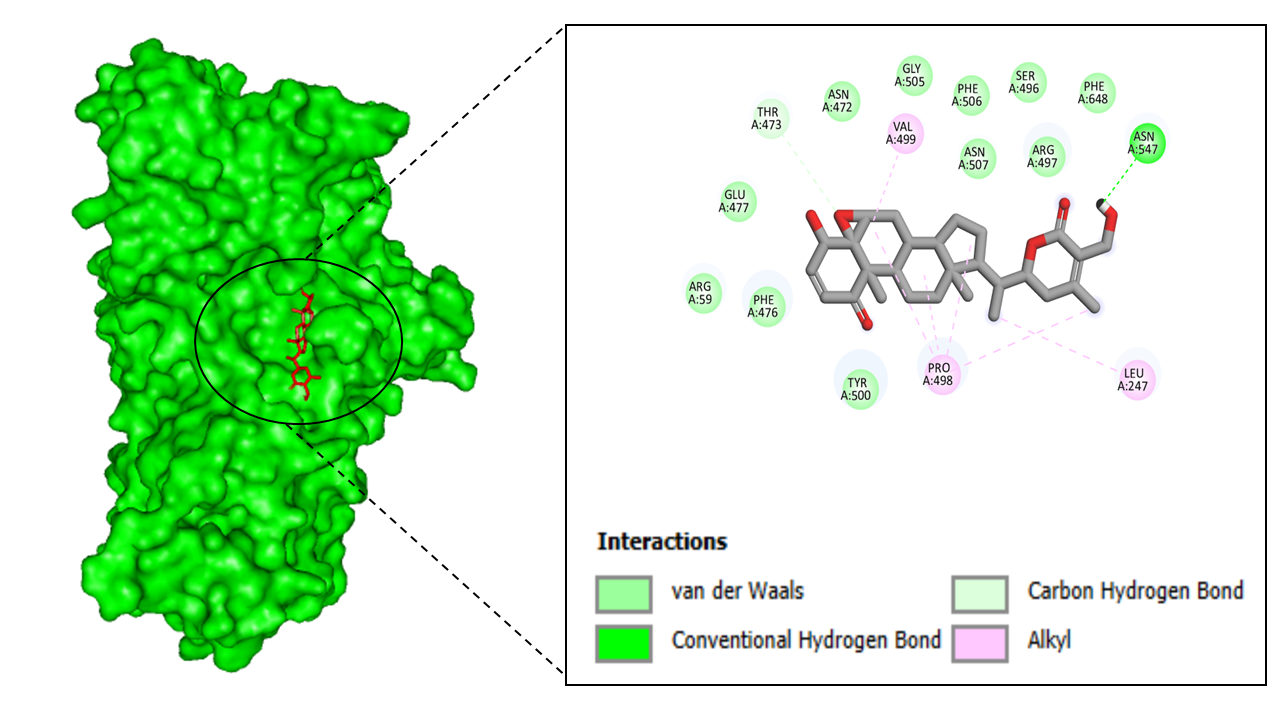

Supplement: Supplementary file 1 — Supplementary Information 1. [file 41598_2025_95163_MOESM1_ESM.zip › docking/Top-2/Docking-37.png]

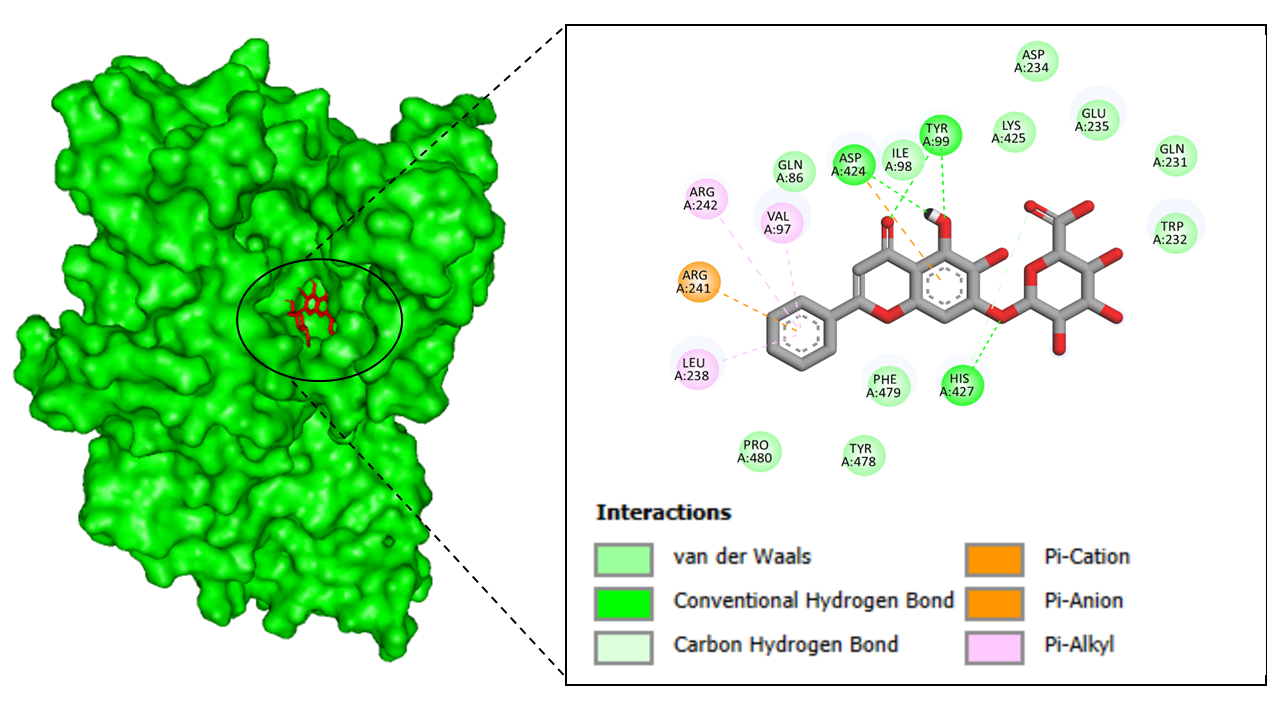

Supplement: Supplementary file 1 — Supplementary Information 1. [file 41598_2025_95163_MOESM1_ESM.zip › docking/Top-2/Docking-82.png]

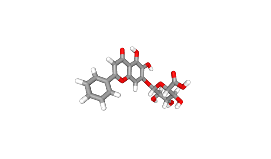

Supplement: Supplementary file 1 — Supplementary Information 1. [file 41598_2025_95163_MOESM1_ESM.zip › Ligands/Baicalin.png]

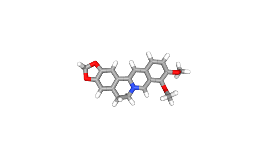

Supplement: Supplementary file 1 — Supplementary Information 1. [file 41598_2025_95163_MOESM1_ESM.zip › Ligands/Berberine.png]

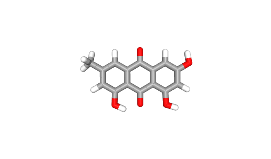

Supplement: Supplementary file 1 — Supplementary Information 1. [file 41598_2025_95163_MOESM1_ESM.zip › Ligands/Emodin.png]

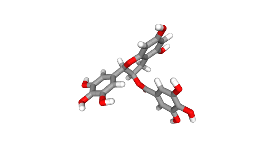

Supplement: Supplementary file 1 — Supplementary Information 1. [file 41598_2025_95163_MOESM1_ESM.zip › Ligands/Epigallocatechin-Gallate.png]

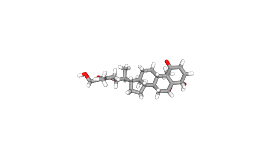

Supplement: Supplementary file 1 — Supplementary Information 1. [file 41598_2025_95163_MOESM1_ESM.zip › Ligands/Withaferin-A.png]

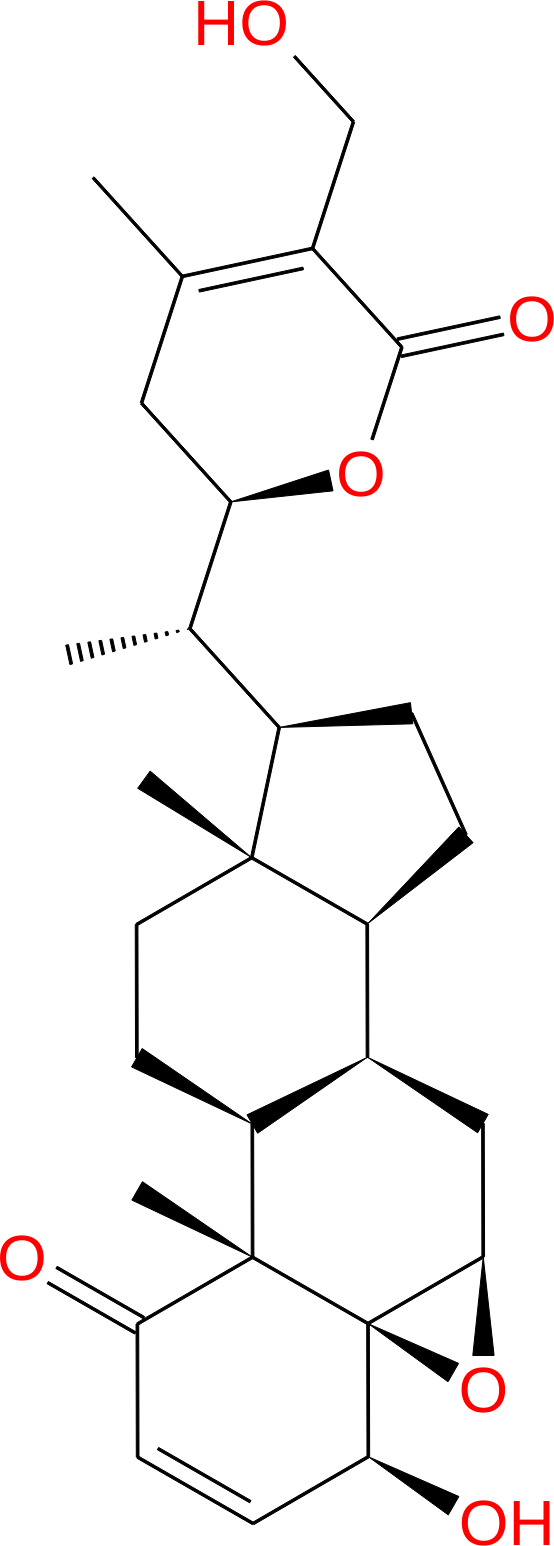

Supplement: Supplementary file 1 — Supplementary Information 1. [file 41598_2025_95163_MOESM1_ESM.zip › MDS-100-results/MDS-100-results/data1/images/L_2d_main.png]

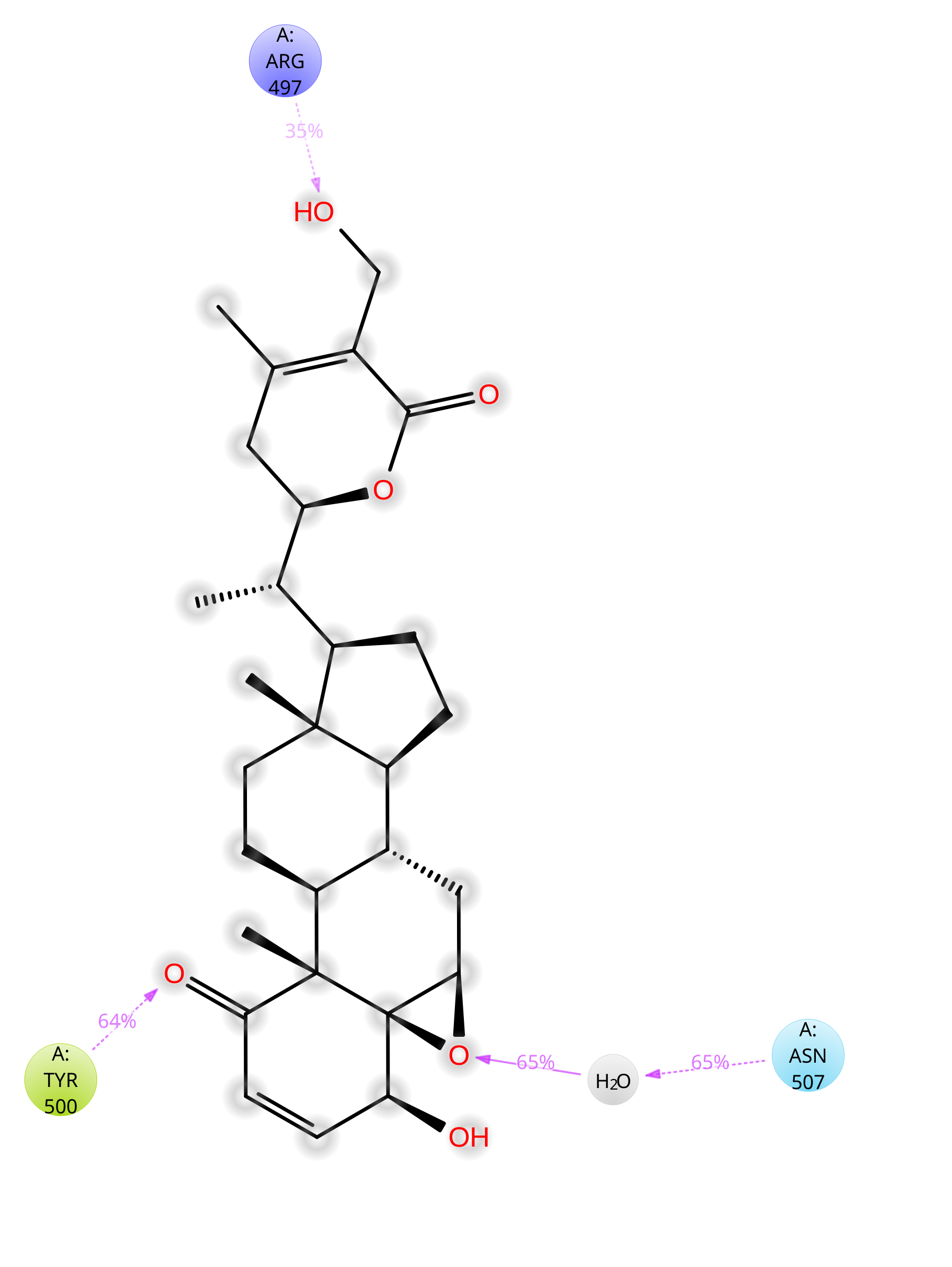

Supplement: Supplementary file 1 — Supplementary Information 1. [file 41598_2025_95163_MOESM1_ESM.zip › MDS-100-results/MDS-100-results/data1/images/LP-Contacts_2d-Summary.png]

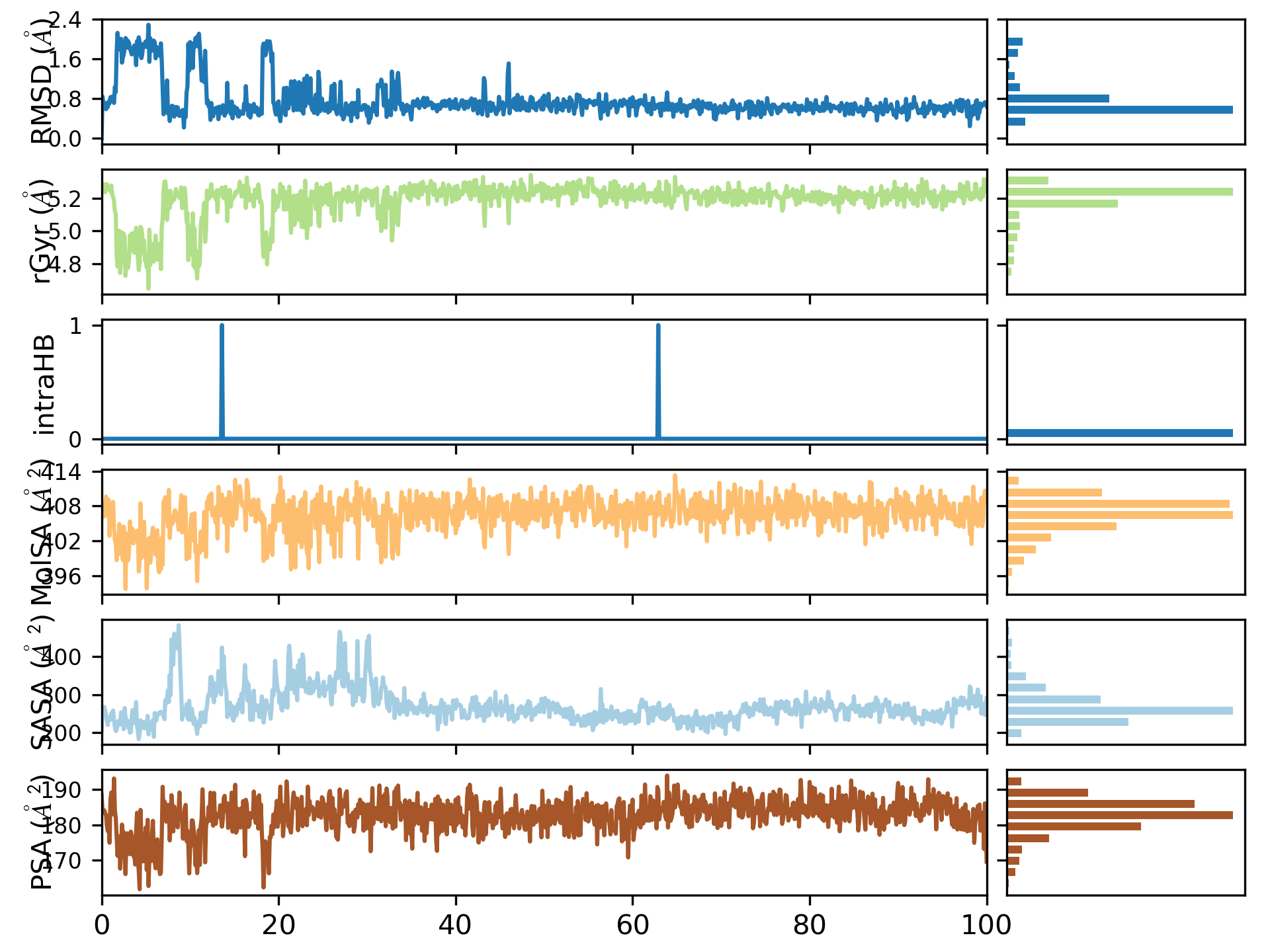

Supplement: Supplementary file 1 — Supplementary Information 1. [file 41598_2025_95163_MOESM1_ESM.zip › MDS-100-results/MDS-100-results/data1/images/L-Properties.png]

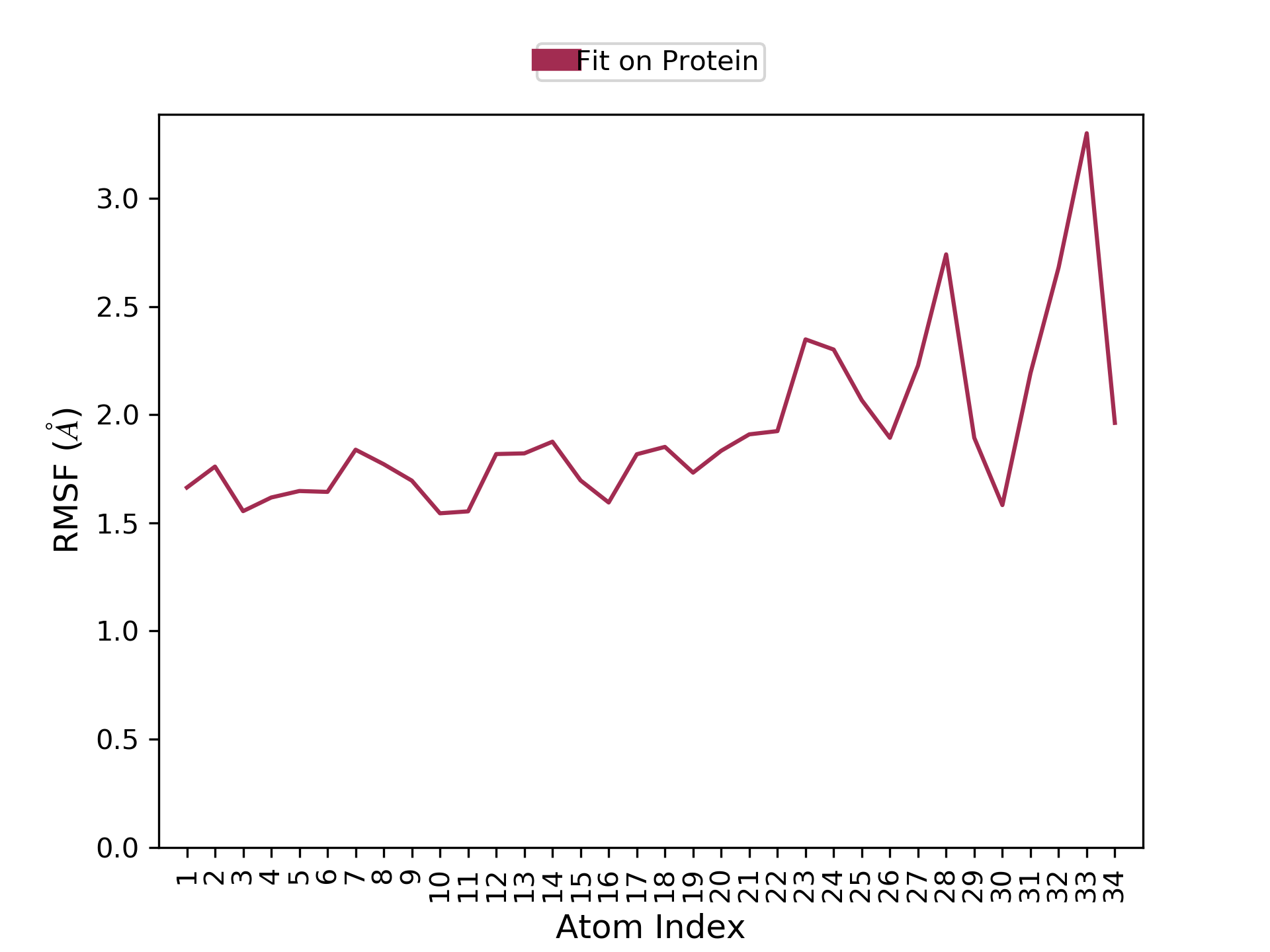

Supplement: Supplementary file 1 — Supplementary Information 1. [file 41598_2025_95163_MOESM1_ESM.zip › MDS-100-results/MDS-100-results/data1/images/L-RMSF.png]

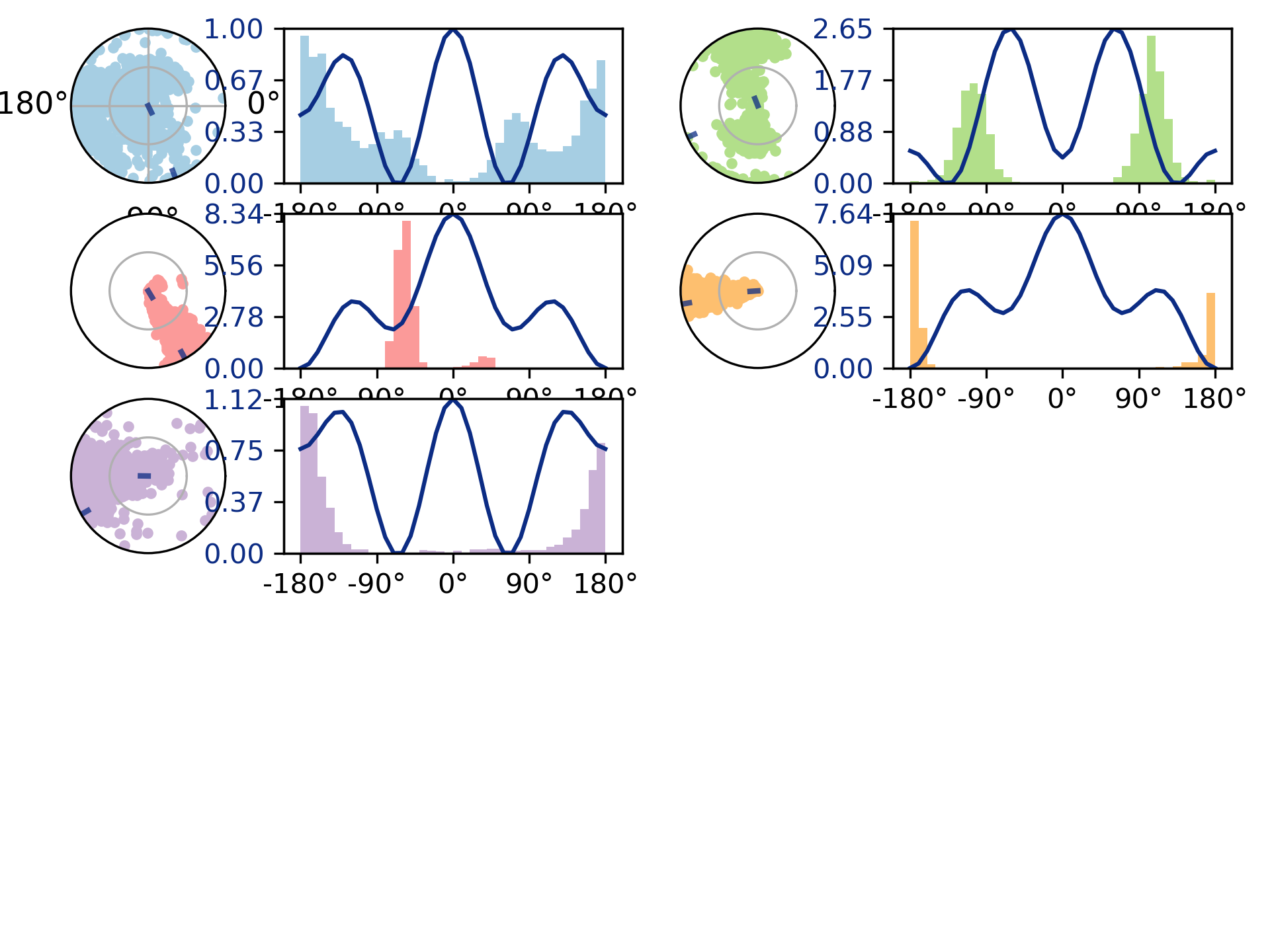

Supplement: Supplementary file 1 — Supplementary Information 1. [file 41598_2025_95163_MOESM1_ESM.zip › MDS-100-results/MDS-100-results/data1/images/L-Torsions.png]

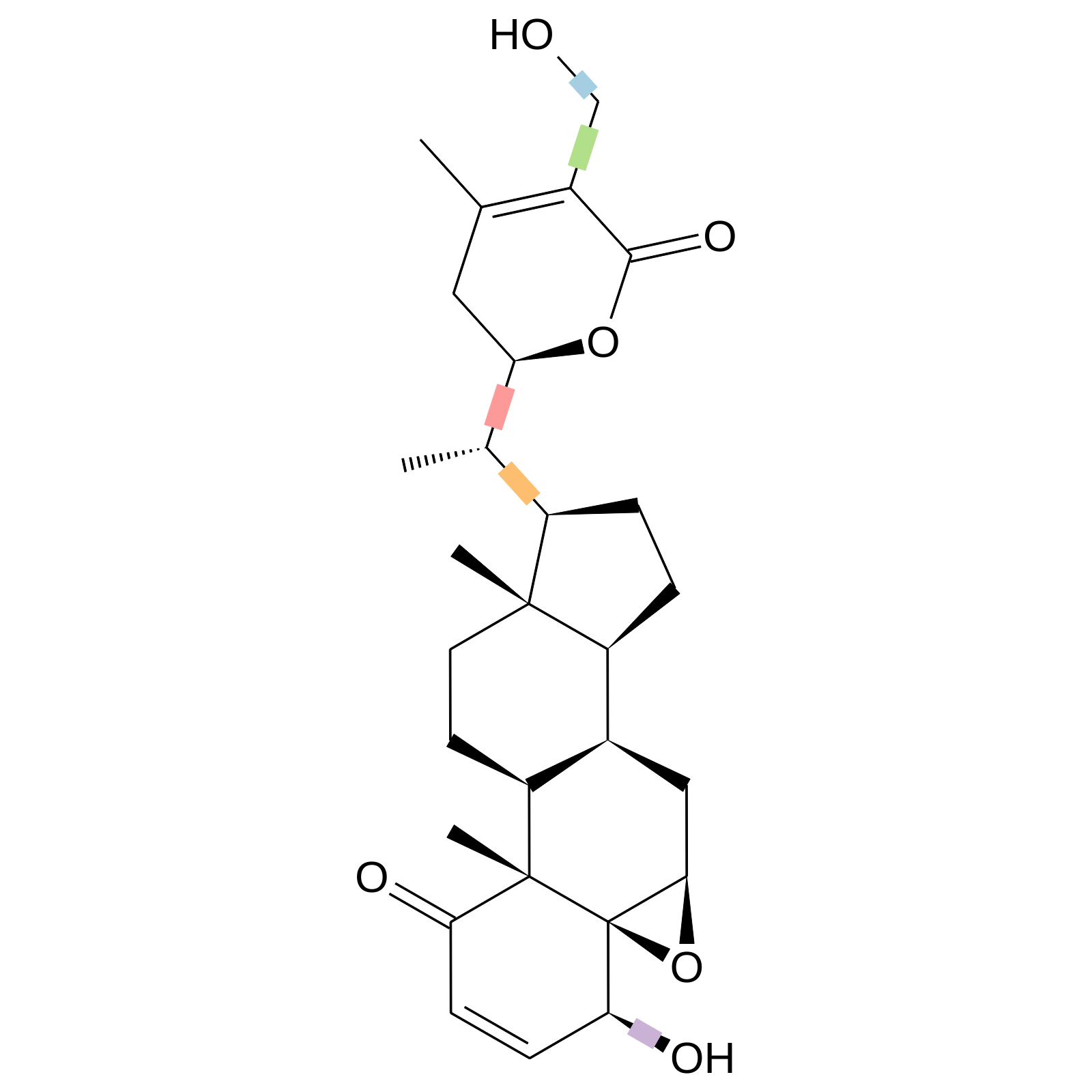

Supplement: Supplementary file 1 — Supplementary Information 1. [file 41598_2025_95163_MOESM1_ESM.zip › MDS-100-results/MDS-100-results/data1/images/L-Torsions-2d.png]

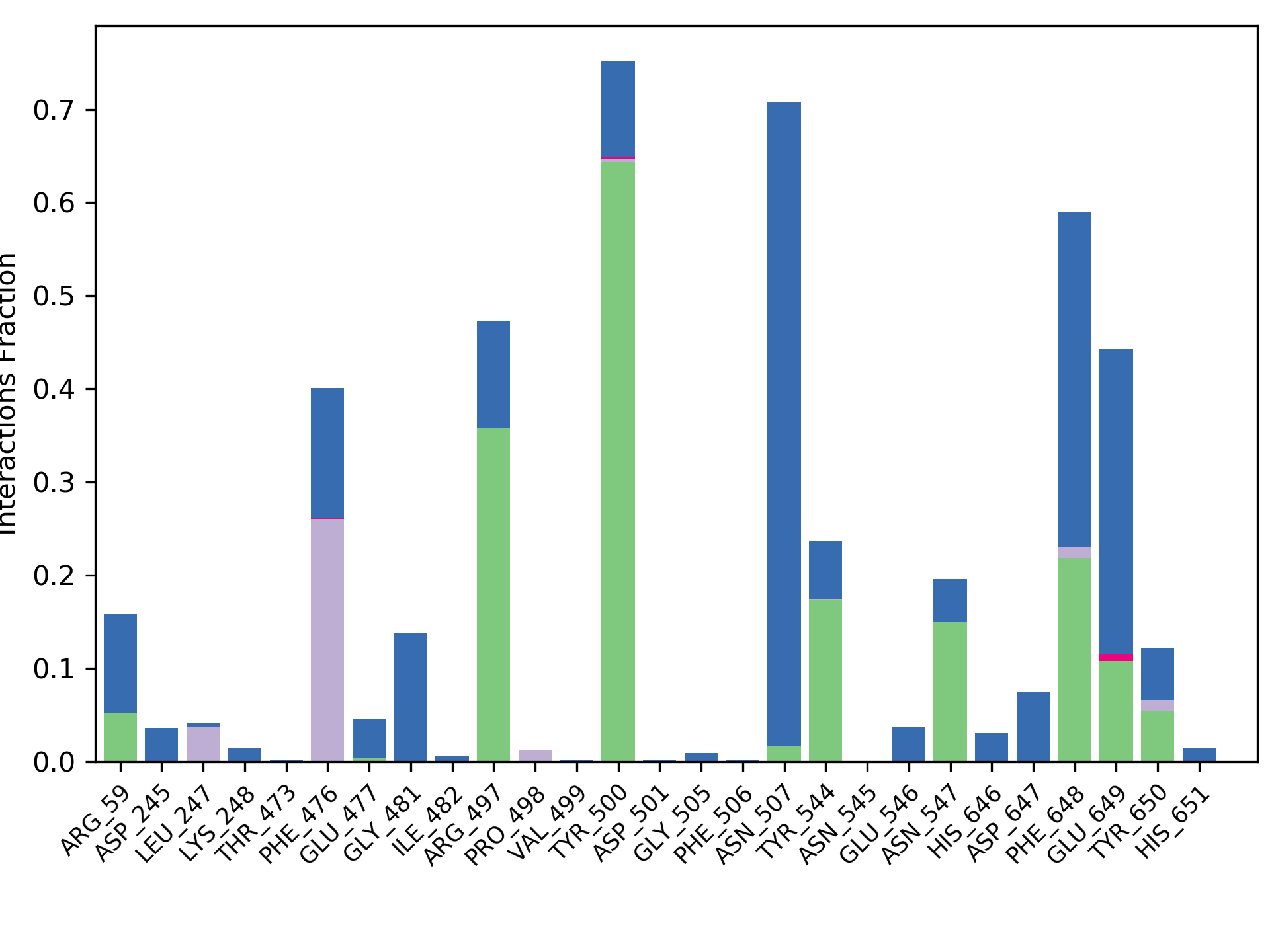

Supplement: Supplementary file 1 — Supplementary Information 1. [file 41598_2025_95163_MOESM1_ESM.zip › MDS-100-results/MDS-100-results/data1/images/PL-Contacts_Histogram.png]

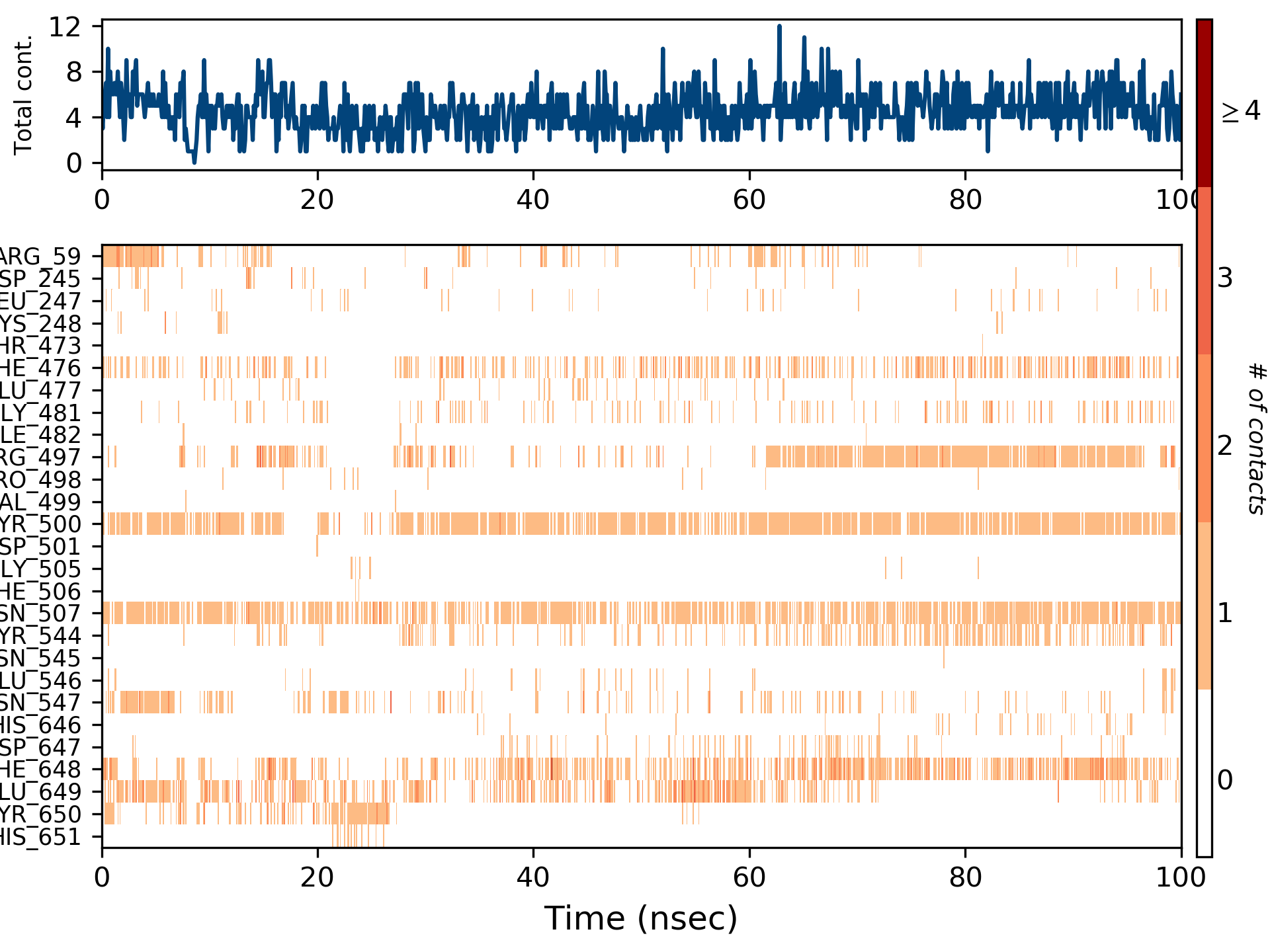

Supplement: Supplementary file 1 — Supplementary Information 1. [file 41598_2025_95163_MOESM1_ESM.zip › MDS-100-results/MDS-100-results/data1/images/PL-Contacts_Timeline.png]

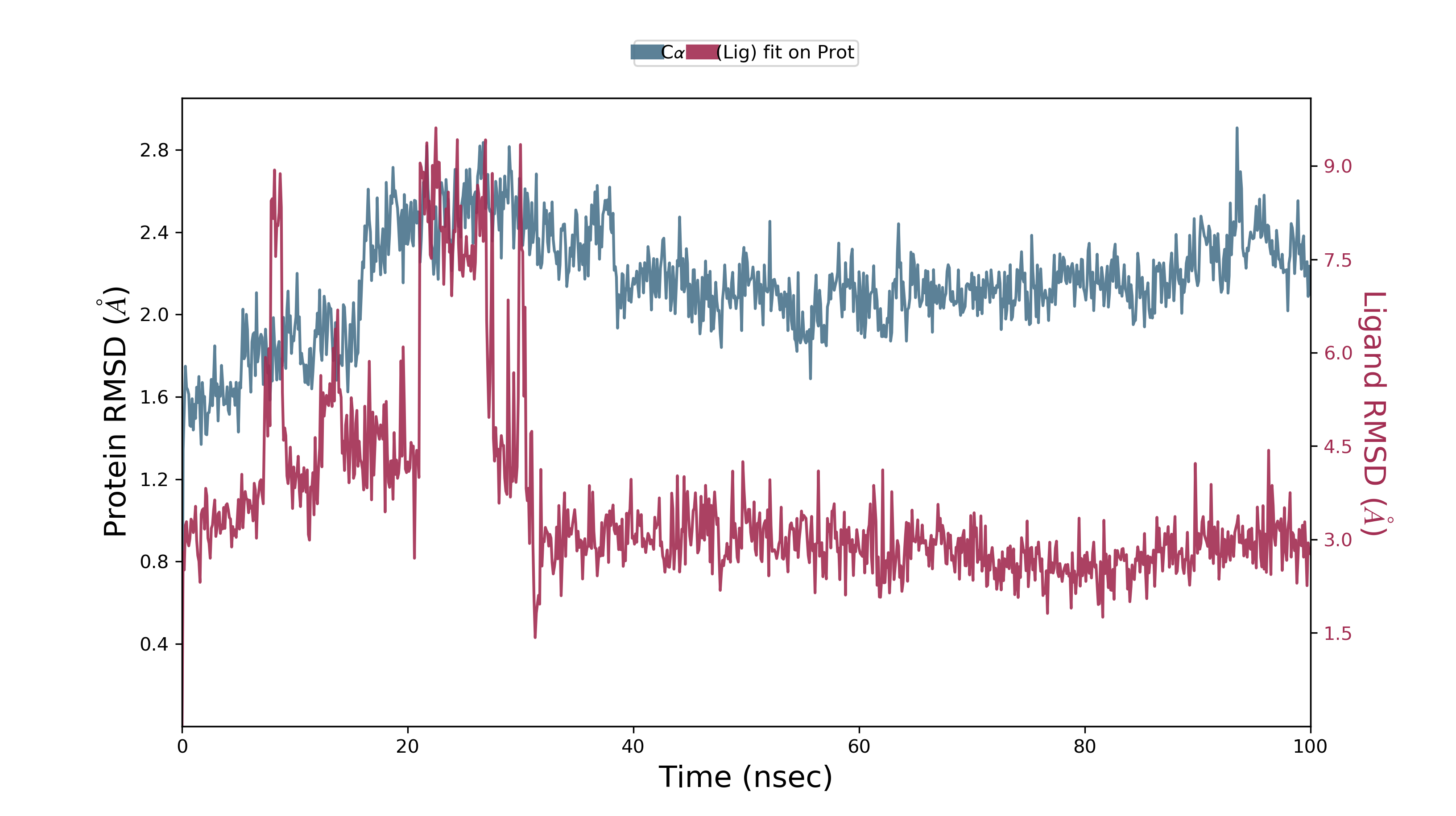

Supplement: Supplementary file 1 — Supplementary Information 1. [file 41598_2025_95163_MOESM1_ESM.zip › MDS-100-results/MDS-100-results/data1/images/PL-RMSD.png]

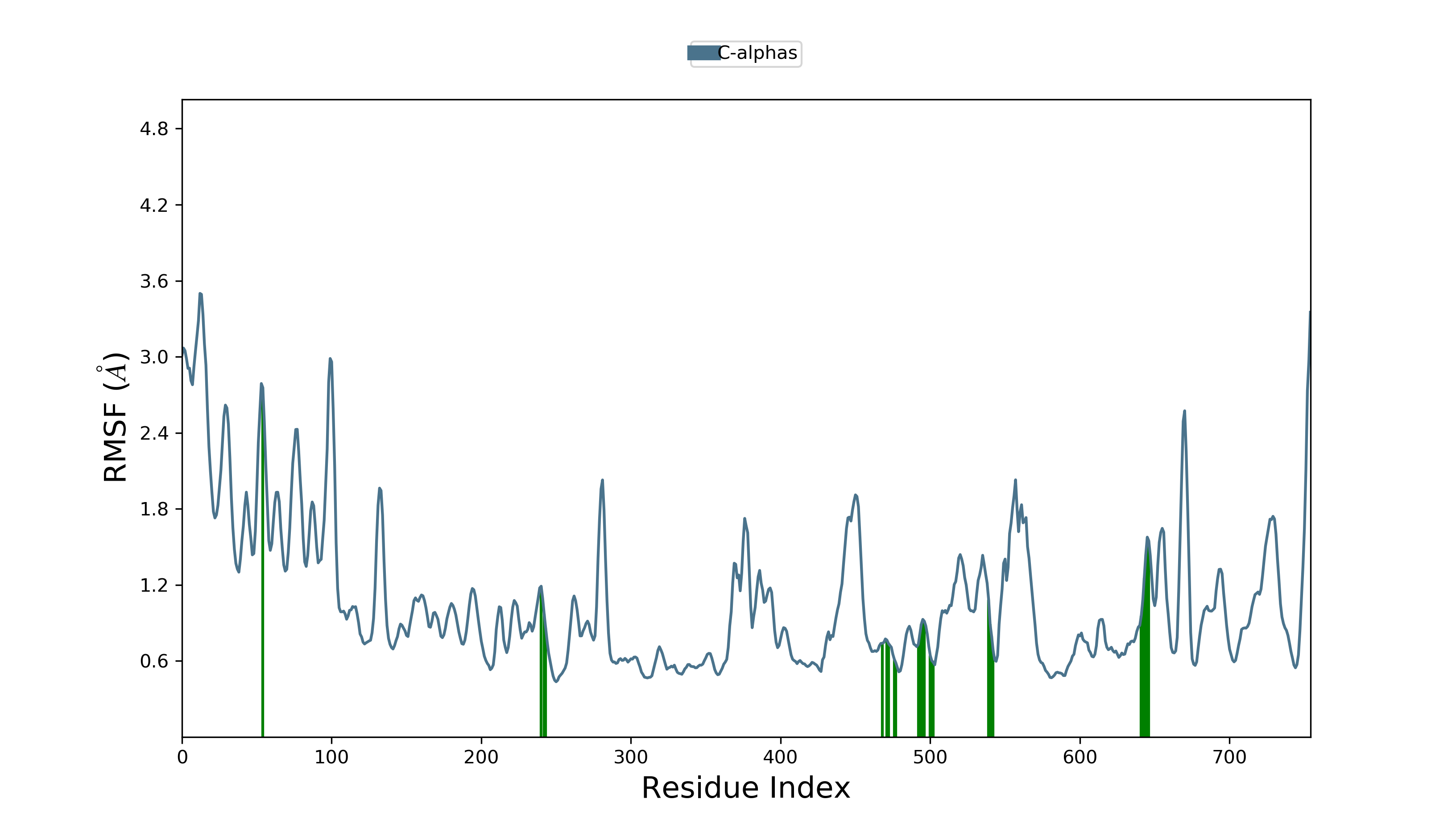

Supplement: Supplementary file 1 — Supplementary Information 1. [file 41598_2025_95163_MOESM1_ESM.zip › MDS-100-results/MDS-100-results/data1/images/P-RMSF.png]

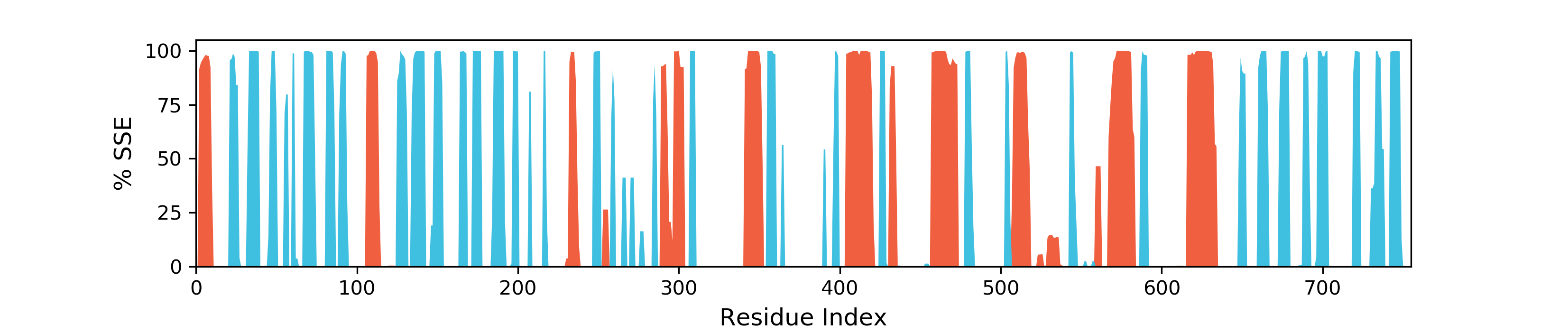

Supplement: Supplementary file 1 — Supplementary Information 1. [file 41598_2025_95163_MOESM1_ESM.zip › MDS-100-results/MDS-100-results/data1/images/P-SSE_Histogram.png]

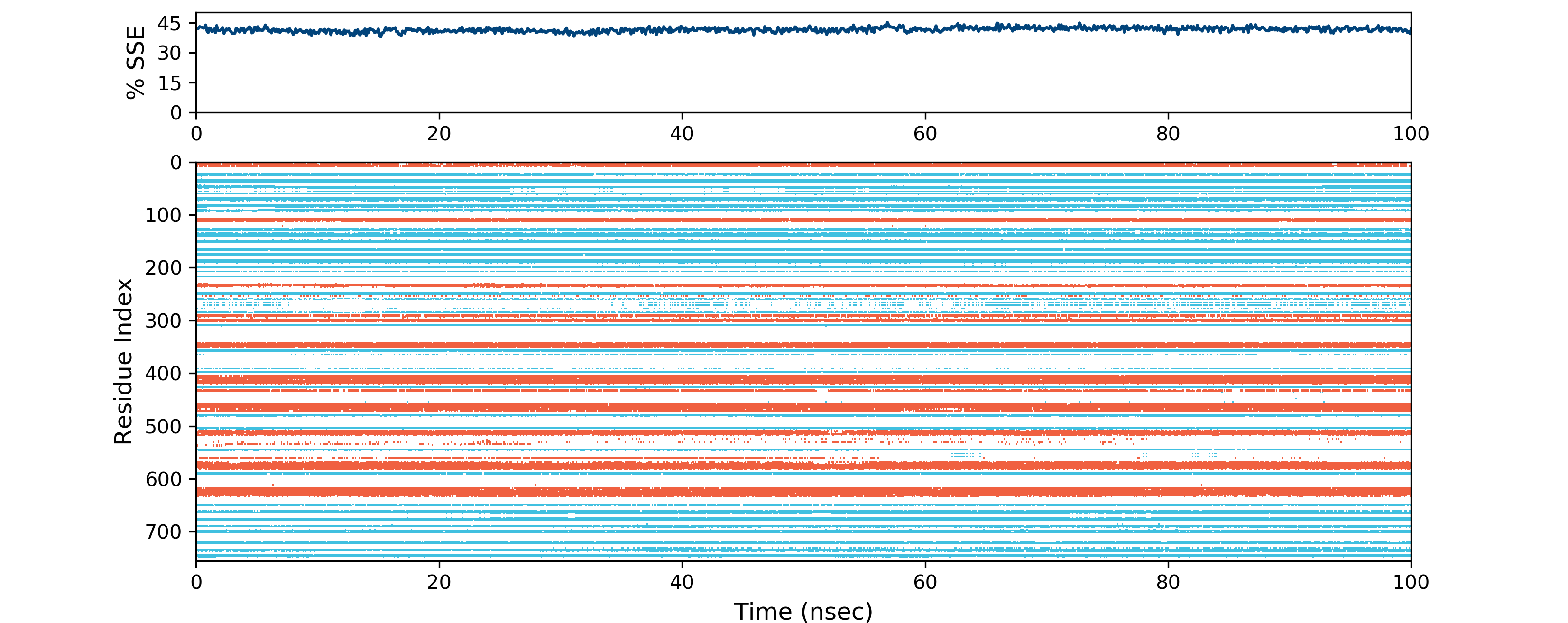

Supplement: Supplementary file 1 — Supplementary Information 1. [file 41598_2025_95163_MOESM1_ESM.zip › MDS-100-results/MDS-100-results/data1/images/P-SSE_Timeline.png]

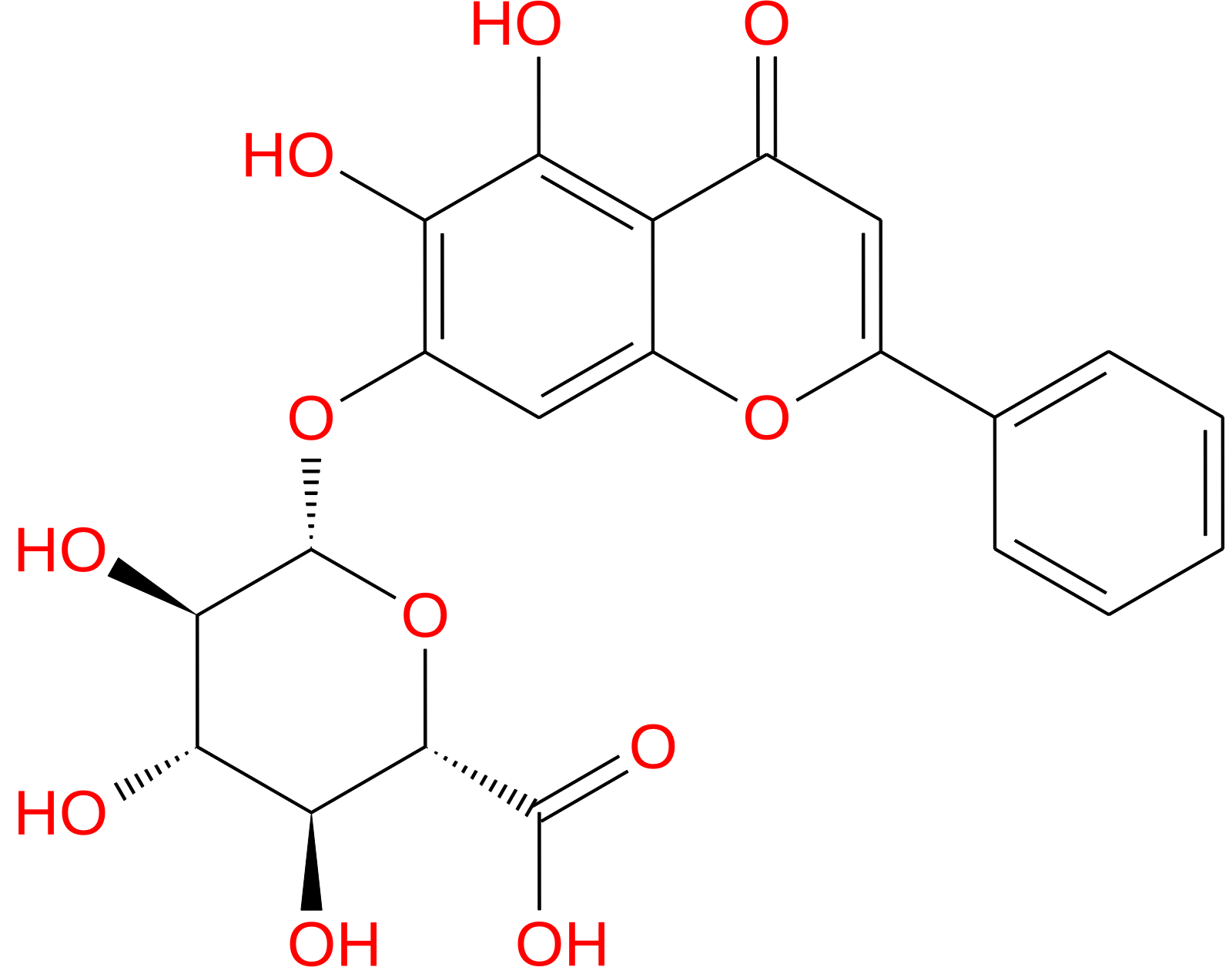

Supplement: Supplementary file 1 — Supplementary Information 1. [file 41598_2025_95163_MOESM1_ESM.zip › MDS-100-results/MDS-100-results/data2/images/L_2d_main.png]

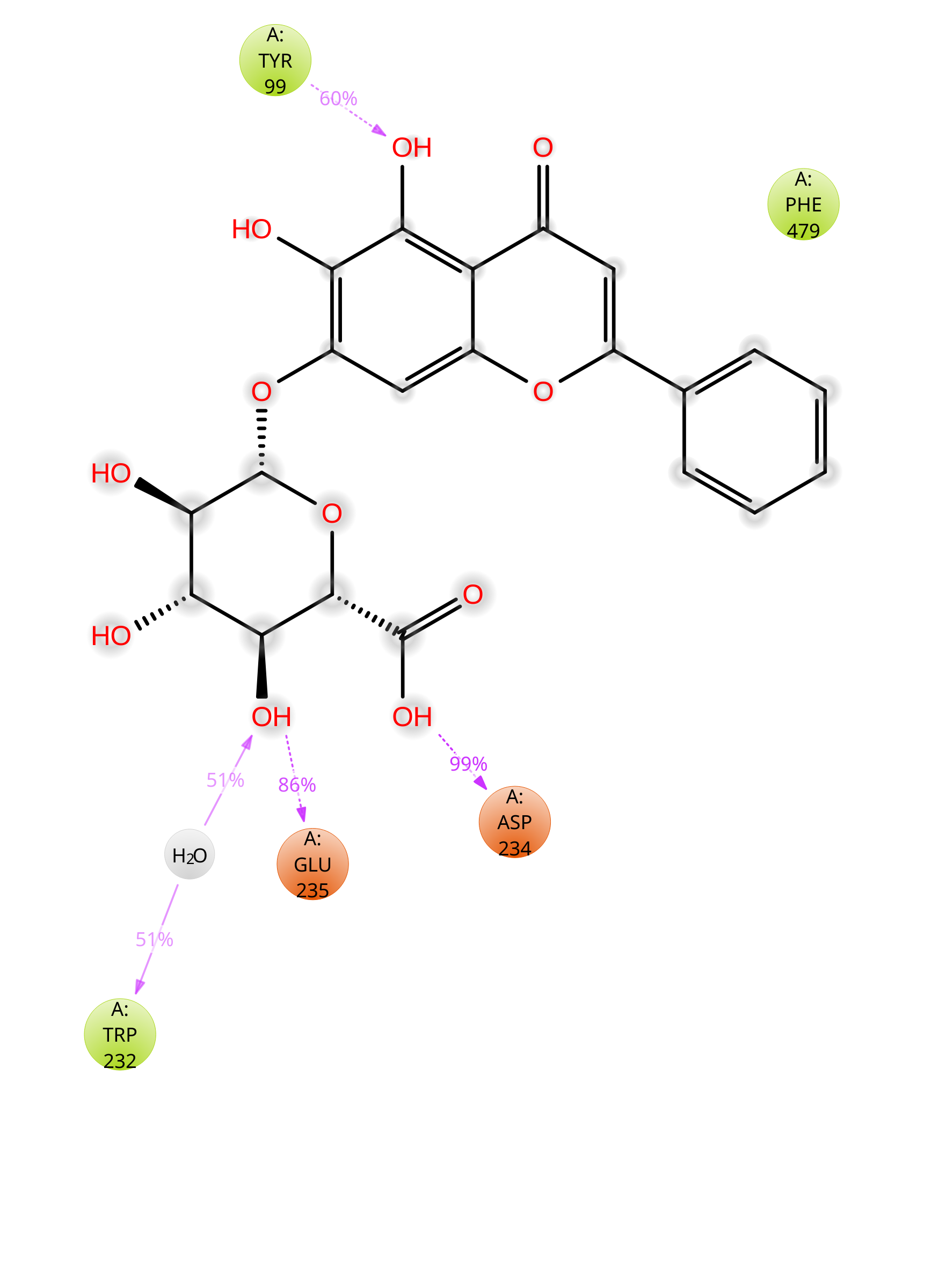

Supplement: Supplementary file 1 — Supplementary Information 1. [file 41598_2025_95163_MOESM1_ESM.zip › MDS-100-results/MDS-100-results/data2/images/LP-Contacts_2d-Summary.png]

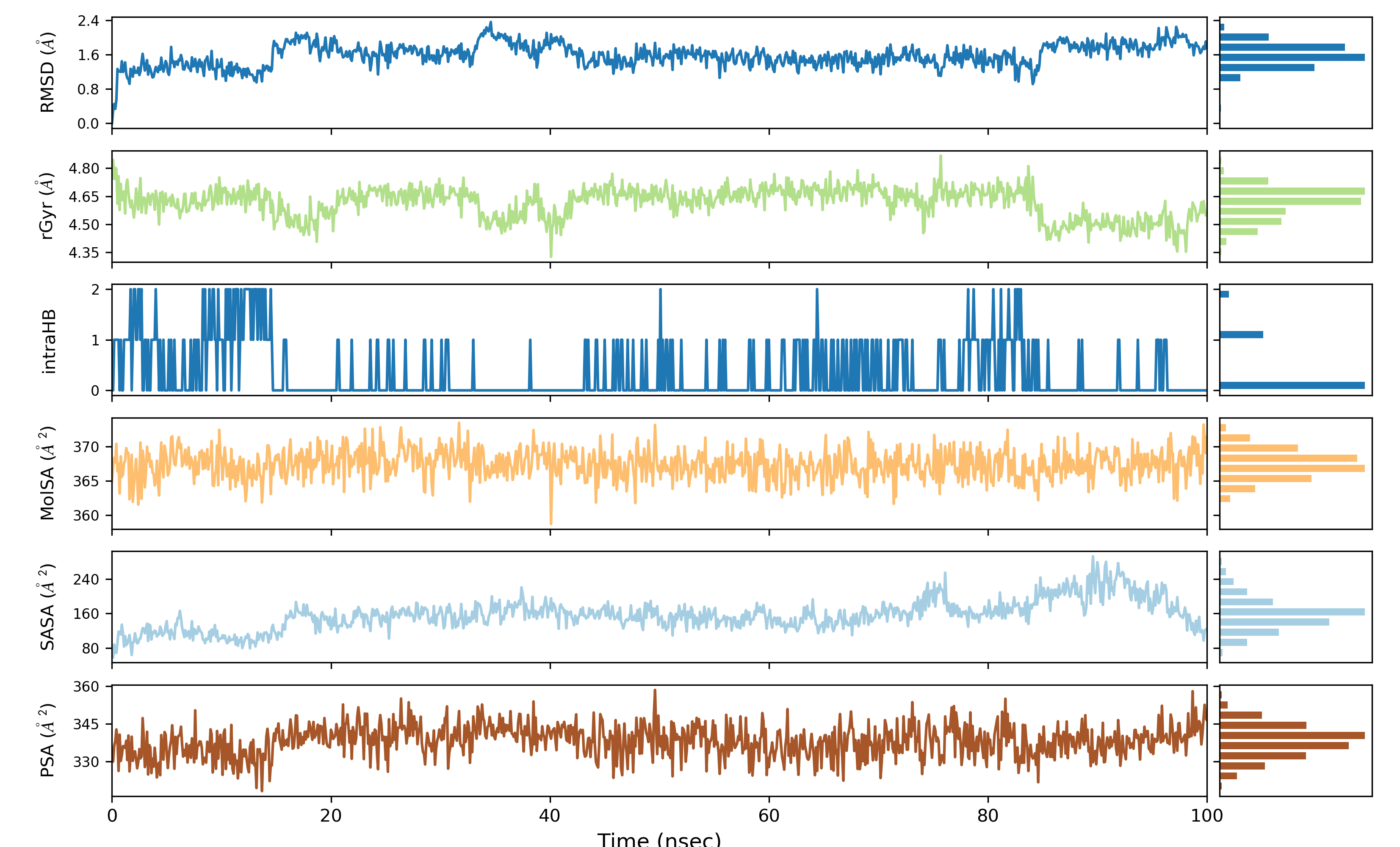

Supplement: Supplementary file 1 — Supplementary Information 1. [file 41598_2025_95163_MOESM1_ESM.zip › MDS-100-results/MDS-100-results/data2/images/L-Properties.png]

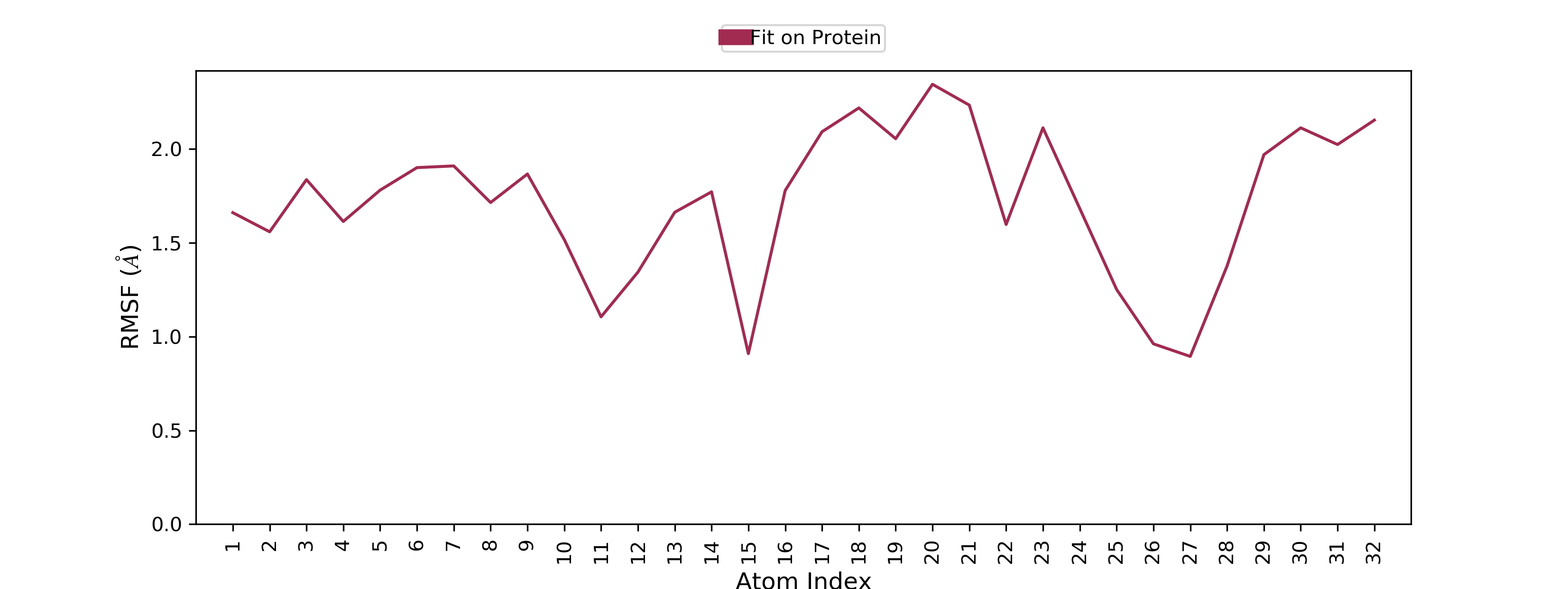

Supplement: Supplementary file 1 — Supplementary Information 1. [file 41598_2025_95163_MOESM1_ESM.zip › MDS-100-results/MDS-100-results/data2/images/L-RMSF.png]

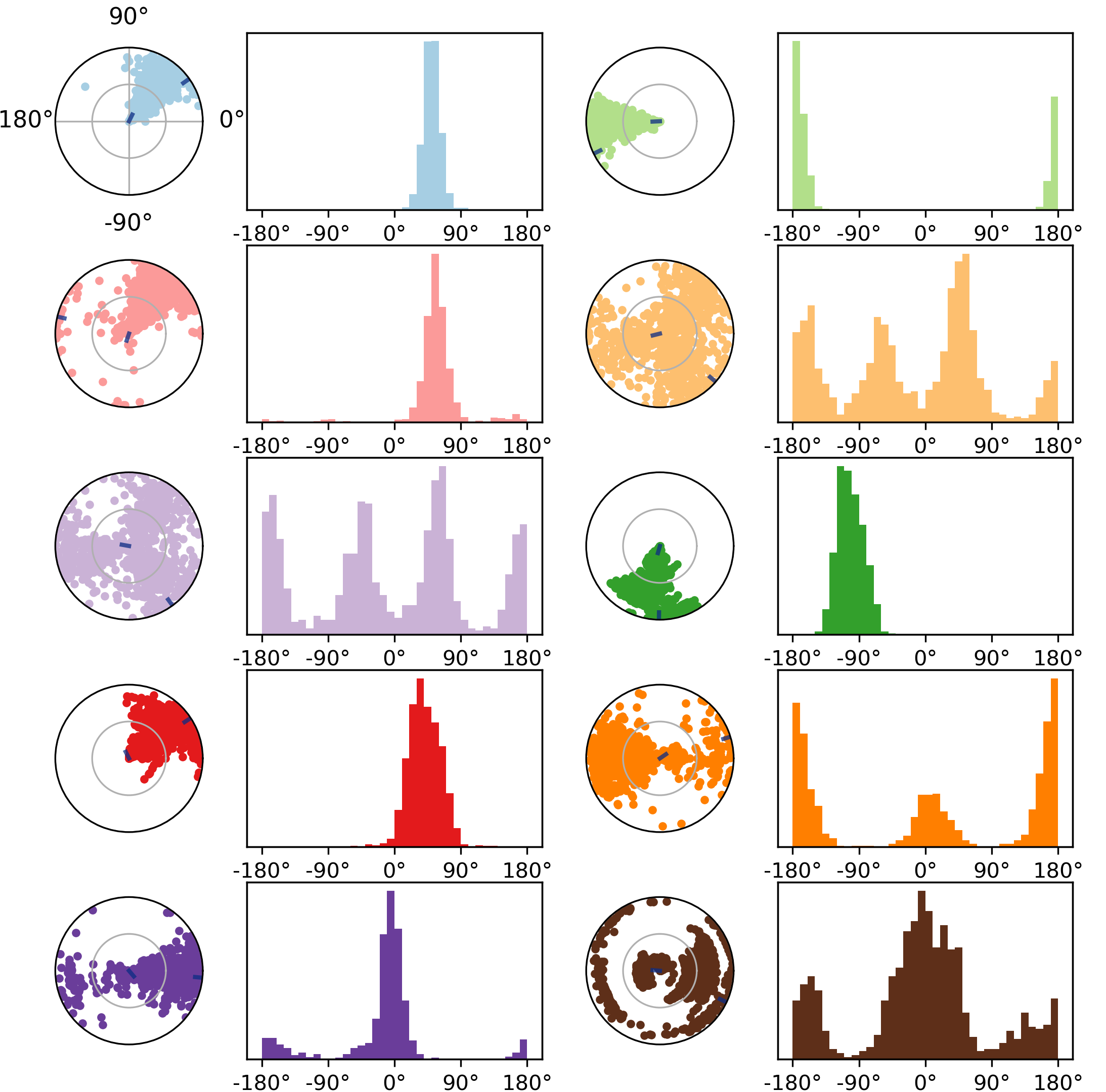

Supplement: Supplementary file 1 — Supplementary Information 1. [file 41598_2025_95163_MOESM1_ESM.zip › MDS-100-results/MDS-100-results/data2/images/L-Torsions.png]

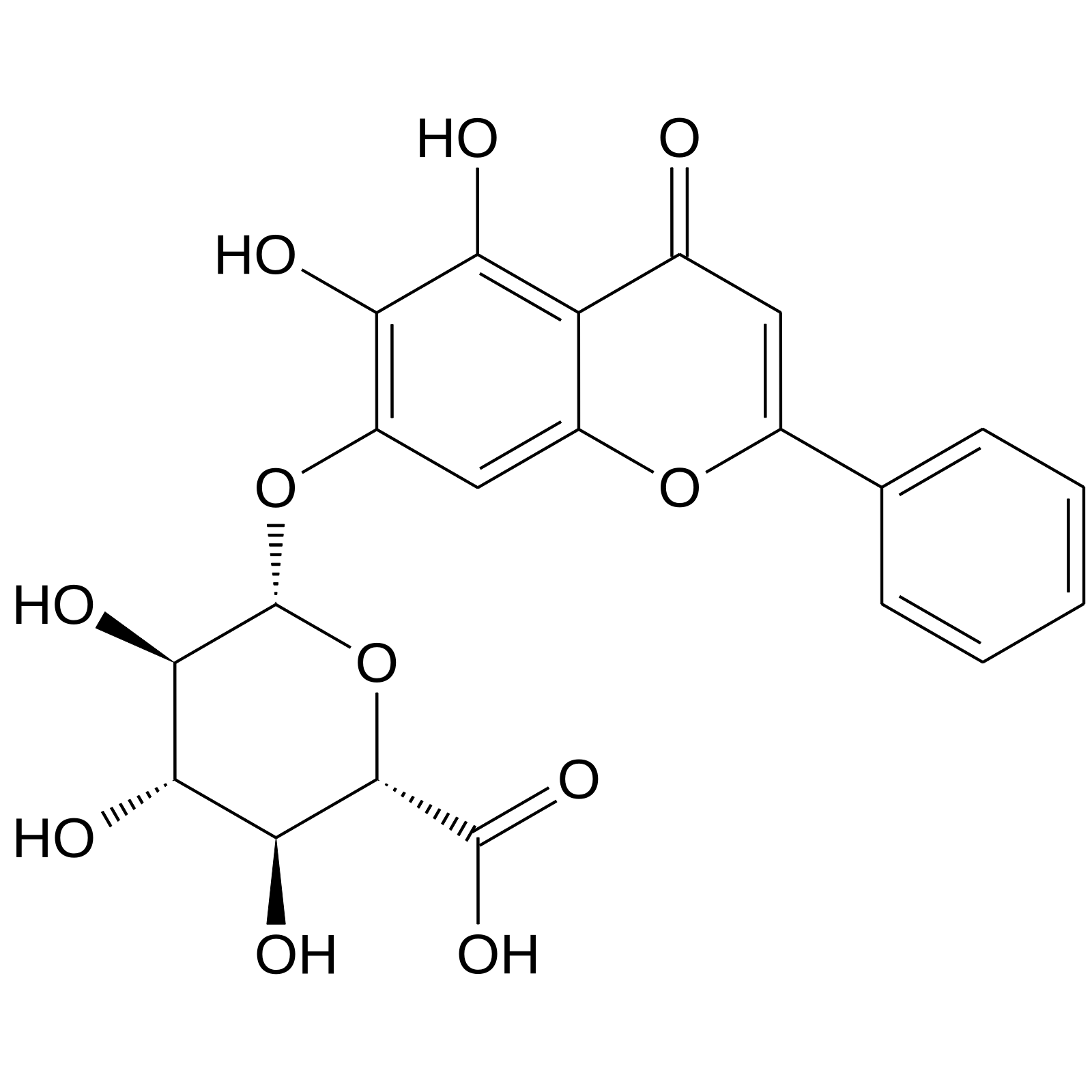

Supplement: Supplementary file 1 — Supplementary Information 1. [file 41598_2025_95163_MOESM1_ESM.zip › MDS-100-results/MDS-100-results/data2/images/L-Torsions-2d.png]

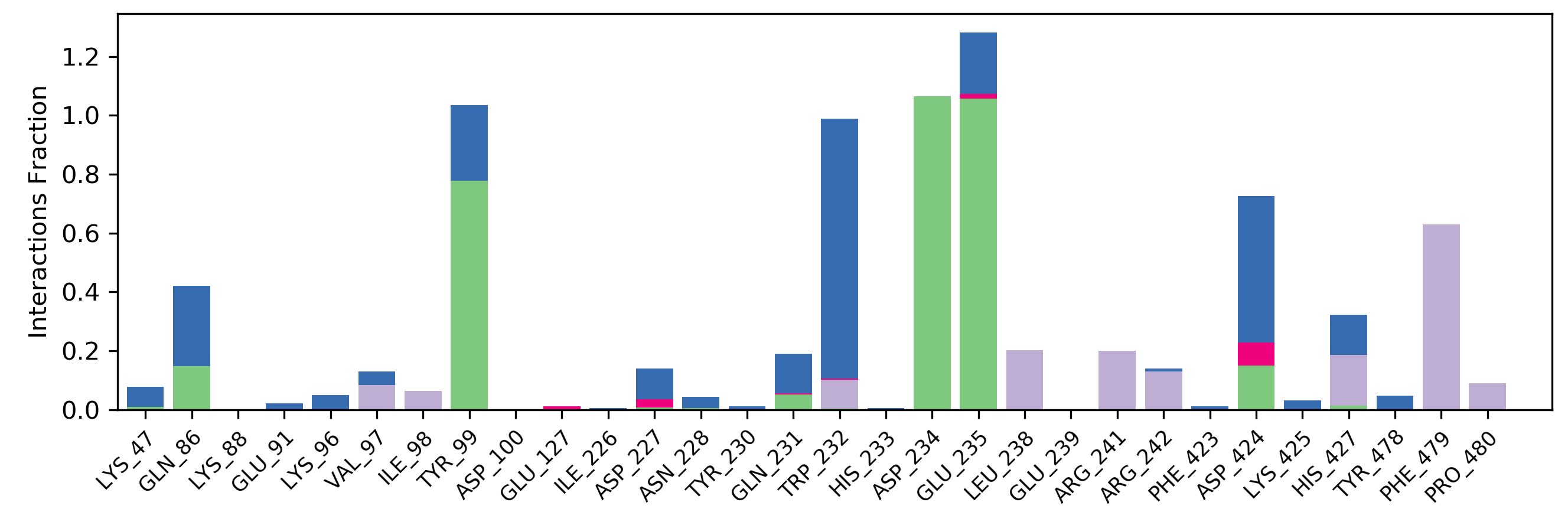

Supplement: Supplementary file 1 — Supplementary Information 1. [file 41598_2025_95163_MOESM1_ESM.zip › MDS-100-results/MDS-100-results/data2/images/PL-Contacts_Histogram.png]

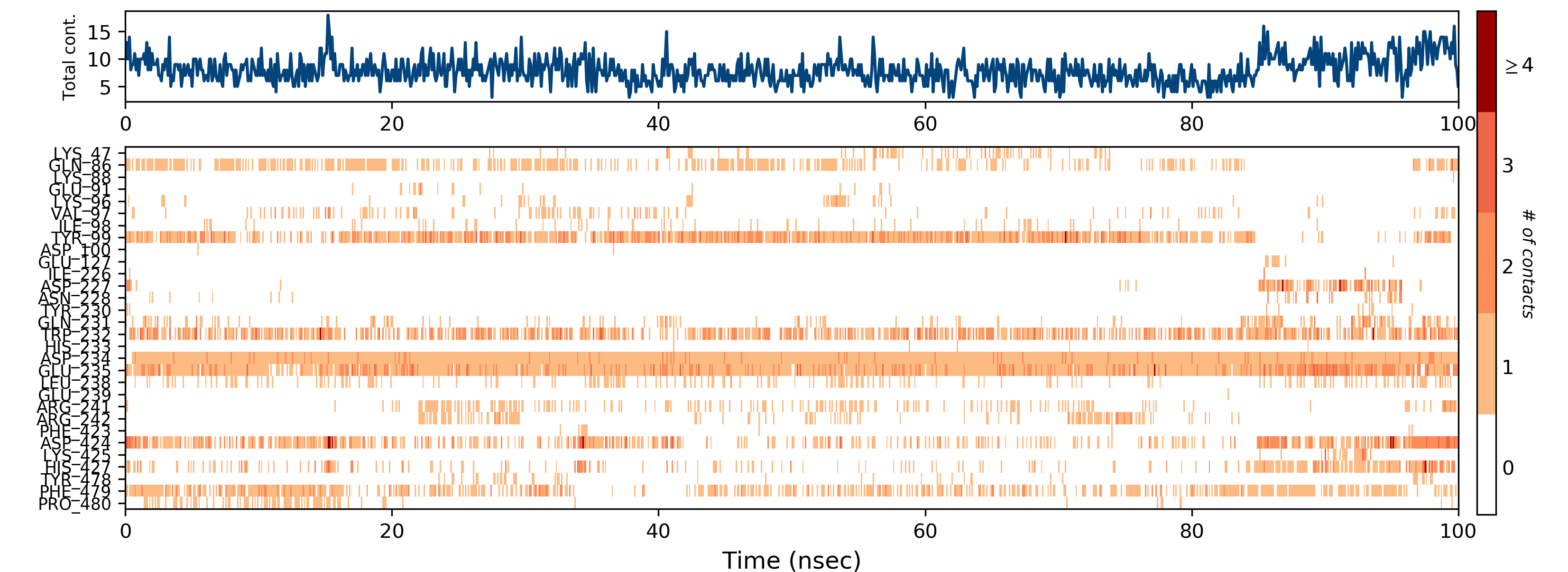

Supplement: Supplementary file 1 — Supplementary Information 1. [file 41598_2025_95163_MOESM1_ESM.zip › MDS-100-results/MDS-100-results/data2/images/PL-Contacts_Timeline.png]

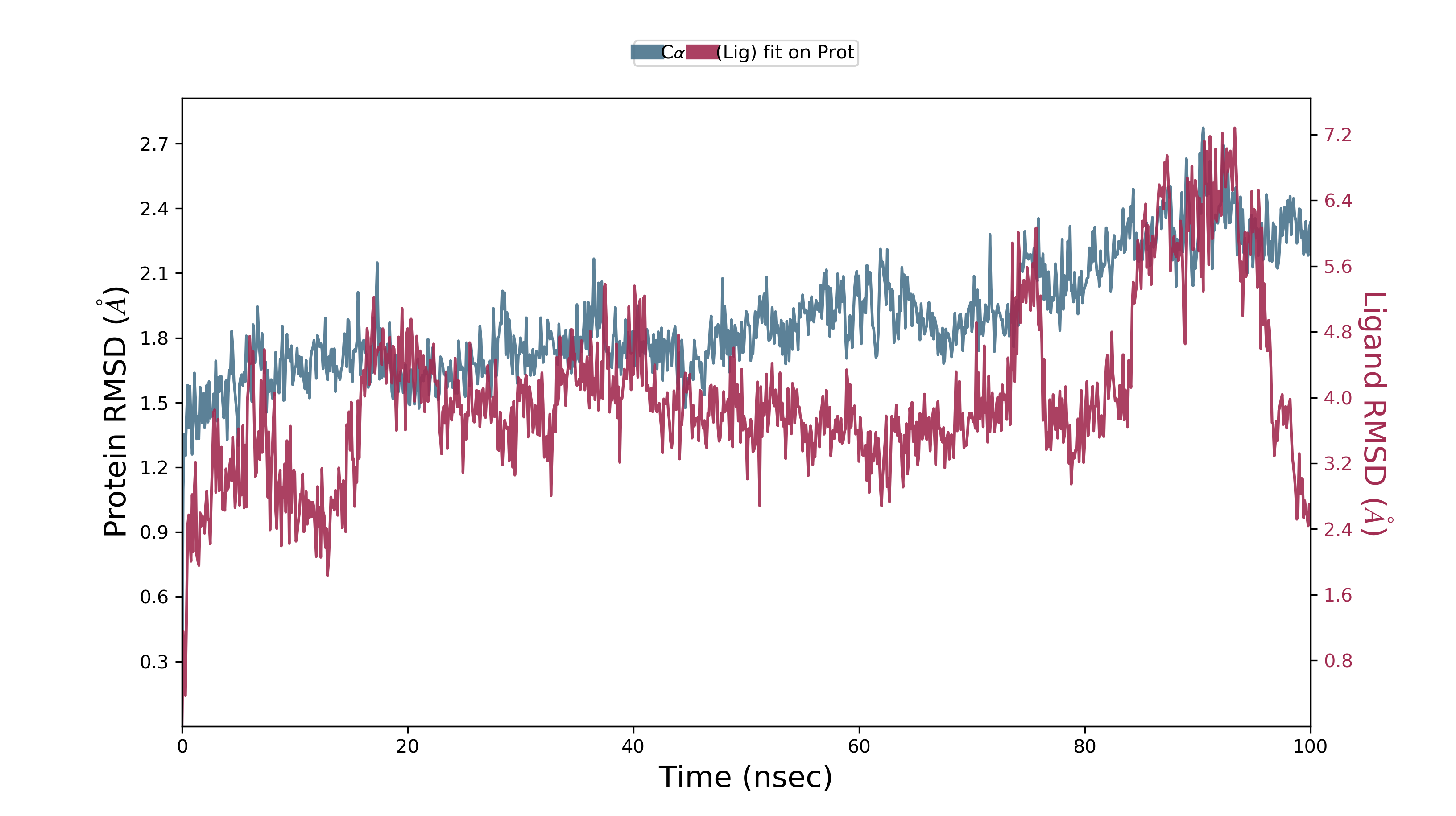

Supplement: Supplementary file 1 — Supplementary Information 1. [file 41598_2025_95163_MOESM1_ESM.zip › MDS-100-results/MDS-100-results/data2/images/PL-RMSD.png]

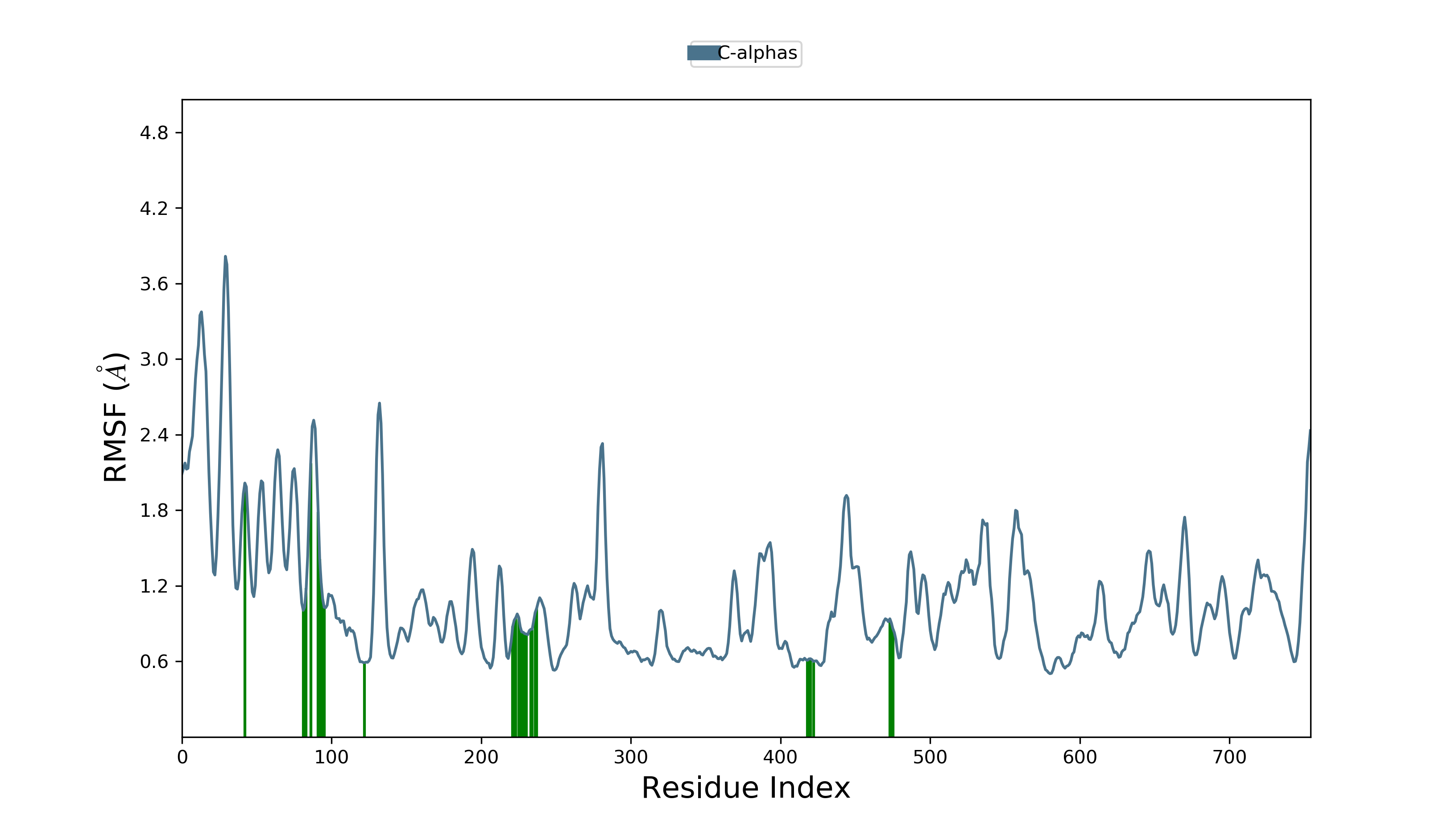

Supplement: Supplementary file 1 — Supplementary Information 1. [file 41598_2025_95163_MOESM1_ESM.zip › MDS-100-results/MDS-100-results/data2/images/P-RMSF.png]

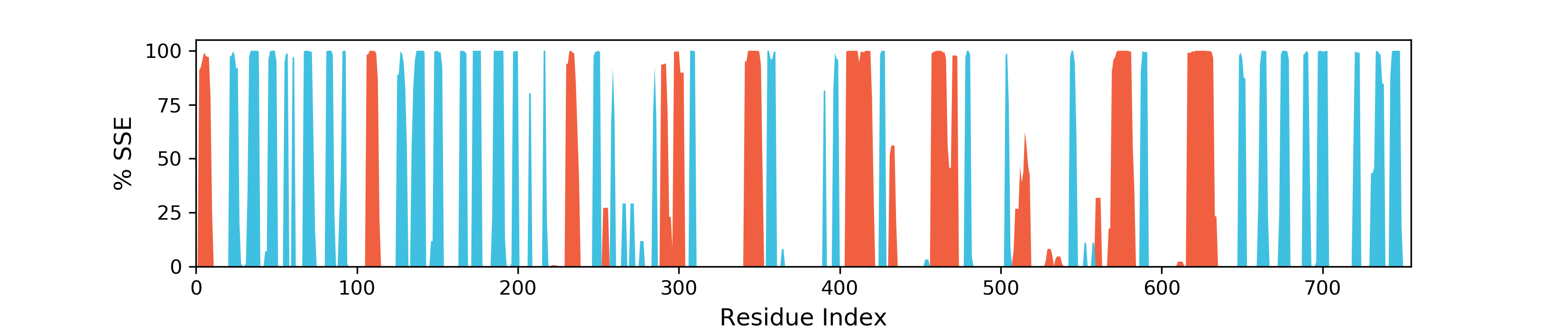

Supplement: Supplementary file 1 — Supplementary Information 1. [file 41598_2025_95163_MOESM1_ESM.zip › MDS-100-results/MDS-100-results/data2/images/P-SSE_Histogram.png]

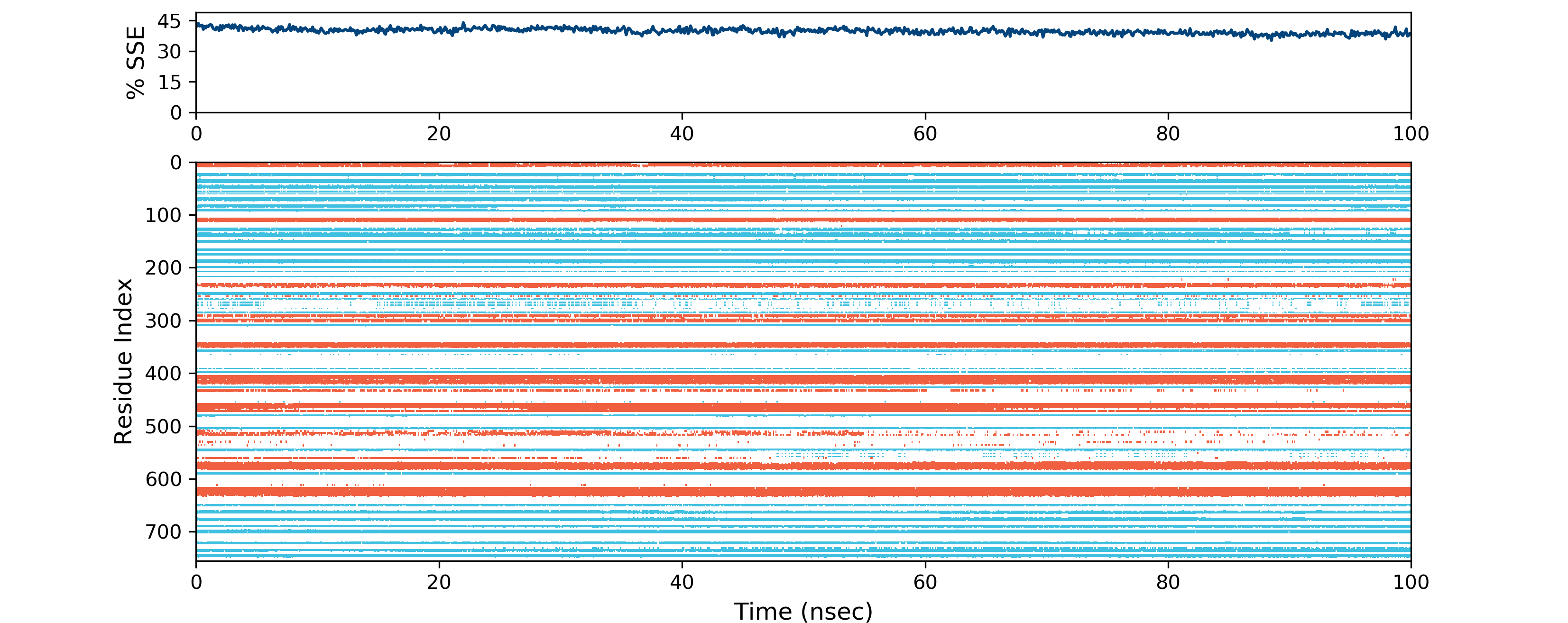

Supplement: Supplementary file 1 — Supplementary Information 1. [file 41598_2025_95163_MOESM1_ESM.zip › MDS-100-results/MDS-100-results/data2/images/P-SSE_Timeline.png]

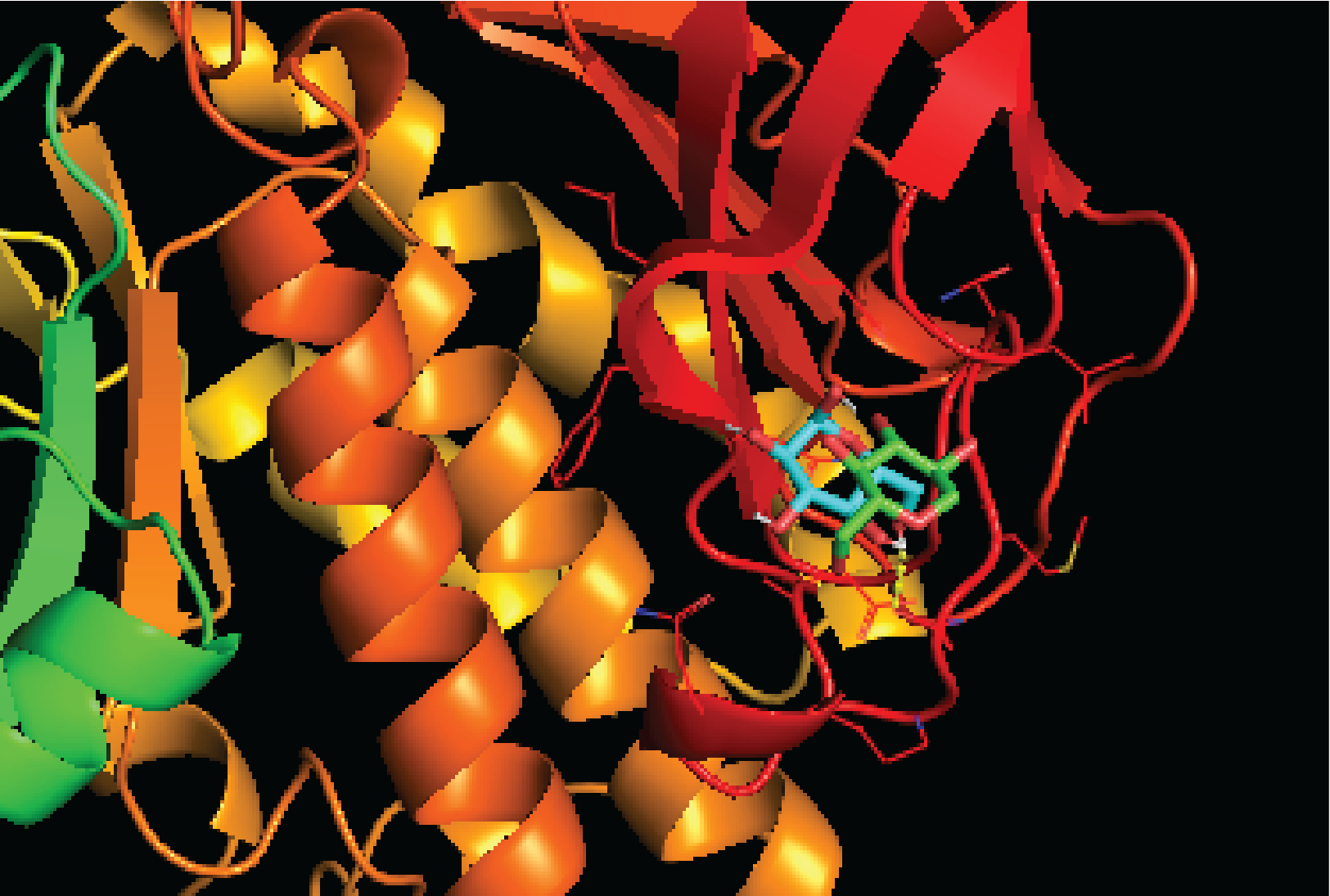

Supplement: Supplementary file 2 — Supplementary Information 2. [file 41598_2025_95163_MOESM2_ESM.zip › Figure S1.png]
