# Supplementary material for: Computational discovery of natural inhibitors targeting enterovirus D68 3C protease using molecular docking pharmacokinetics and dynamics simulations
Source: Sci Rep. 2025 Mar 31;15:11015. doi: 10.1038/s41598-025-95163-y (PMC11958634; doi:10.1038/s41598-025-95163-y)
Supplement: Supplementary file 2 — Supplementary Information 2. [file 41598_2025_95163_MOESM2_ESM.zip › Supplementary Figures.docx]

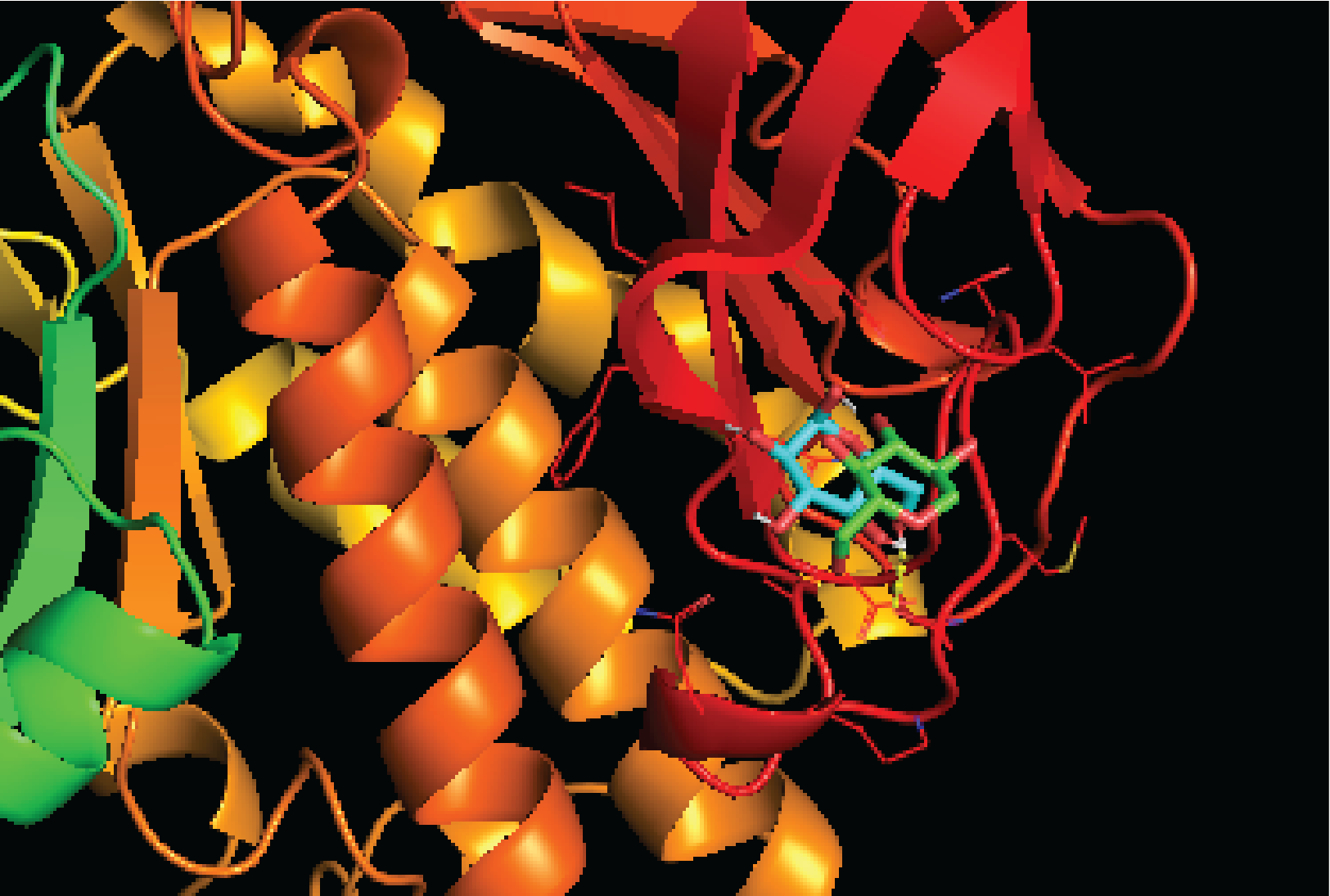


Figure S1. Redocking of the Co-Crystallized Ligand in the Active Site of 3C Protease (PDB ID: 5QGY). The structural alignment of the redocked ligand (cyan) with the original co-crystallized ligand (green) within the active site of the 3C protease is shown. The surrounding protein residues are represented as a ribbon model, with α-helices in orange, β-sheets in red, and loops in green. Key interactions, including hydrogen bonds (dashed lines), stabilize the ligand within the binding pocket. The close overlay of the redocked and crystallographic ligand confirms the accuracy of the docking protocol, with minimal deviation, validating the reliability of the molecular docking approach.
